# Multi-Omics Mechanism of Chronic Gout Arthritis and Discovery of the Thyroid Hormone–AMPK–Taurine Metabolic Axis

**DOI:** 10.3390/cells15010041

**Published:** 2025-12-25

**Authors:** Guizhen Zhu, Yuan Luo, Xiangyi Zheng, Zhusong Mei, Qiao Ye, Jie Peng, Fengsen Duan, Yueying Cui, Peiyu An, Yangqian Song, Hongxia Li, Haitao Zhang, Guangyun Wang

**Affiliations:** 1 Laboratory of Clinical Medicine, Air Force Medical Center, Air Force Medical University, People’s Liberation Army of China, Beijing 100142, China; 13121437811@163.com (G.Z.); ly-navy@163.com (Y.L.); zhengxy12345@163.com (X.Z.); mzsa@163.com (Z.M.); yeqiao333@163.com (Q.Y.); 19991205301@163.com (J.P.); dfs_789@126.com (F.D.); cyygsjyg@163.com (Y.C.); yupeipyjj@sina.com (P.A.); 2 Rheumatology and Immunology Department, Air Force Medical Center, Air Force Medical University, People’s Liberation Army of China, Beijing 100142, China; m15168405793@163.com (Y.S.); hxli2005@126.com (H.L.); 3 Cardiovascular Department, Air Force Medical Center, Air Force Medical University, People’s Liberation Army of China, Beijing 100142, China

**Keywords:** acute gouty arthritis, chronic gouty arthritis, multi-omics integrative analysis, pathogenesis, biomarkers

## Abstract

**Highlights:**

**What are the main findings?**

- Multi-omics profiling reveals nine persistently dysregulated proteins and 11 consistently altered metabolites during the transition from acute to chronic gouty arthritis.
- Chronic gout development involves significant perturbations in key pathways—thyroid hormone synthesis, AMPK signaling, and taurine metabolism—and a concomitant shift in the immune response from acute activation to chronic inflammation.

**What are the implications of the main finding?**

- The study identifies a coordinated disruption of the thyroid hormone–AMPK–taurine metabolic axis and immune microenvironment remodeling as central to chronic gout progression.
- These findings offer potential targets for early diagnosis and targeted interventions to prevent irreversible joint damage in chronic gouty arthritis.

**Abstract:**

The acute gouty arthritis (AGA) to chronic gouty arthritis (CGA) transition is a critical phase leading to irreversible joint damage and systemic complications. However, current molecular mechanism investigations have remained limited to single-omics approaches that lack comprehensive multi-omics explorations. We integrate high-depth data-independent acquisition (DIA) proteomics and untargeted metabolomics to analyze serum samples from healthy controls (*n* =28), AGA (*n* = 31), and CGA (*n* = 14) patients to address this gap. Through differential expression analysis, we identified nine persistently dysregulated pivotal proteins with robust discriminative capacity, including the urate excretion regulator ZBTB20 and inflammation/immune-related proteins (GUCY1A2, CNDP1, LYZ, SERPINA5, GSN). Additionally, 11 consistently altered core metabolites with diagnostic potential were detected, indicating perturbations in sex hormones, thyroid hormones, gut microbiota-derived metabolites, environmental exposures, and nutritional factors. Multi-omics KEGG enrichment analysis highlighted thyroid hormone synthesis, AMPK signaling pathway, and taurine and hypotaurine metabolism as central pathways. Correlation network analysis further revealed significant immune dysregulation, illustrating an evolution from acute immune activation to chronic inflammation during AGA-to-CGA progression. Our study establishes that a coordinated disruption of the thyroid hormone–AMPK–taurine metabolic axis and concomitant immune microenvironment remodeling is associated with chronic gout development. These findings provide critical targets for developing early diagnostic indicators and targeted interventions for CGA.

## 1. Introduction

Gout is an inflammatory arthropathy driven by persistent hyperuricemia, with a growing global prevalence [1,2,3]. While acute gouty arthritis (AGA) is well-characterized by NLRP3 inflammasome activation triggered by monosodium urate crystals [4], the molecular mechanisms underlying the transition to chronic gouty arthritis (CGA) remain poorly understood. This chronic stage, marked by joint destruction, tophi, and persistent inflammation, represents a critical transition in gout pathology [5,6,7]. Notably, the weak correlation between serum urate levels and joint damage suggests that systemic metabolic and immune dysregulation, beyond crystal deposition, plays a critical role in disease chronicity [8].

Recent advances in omics technologies have provided new avenues for deciphering the mechanisms of gout chronicity [9,10,11]. Proteomics allows the systematic profiling of protein expression, modifications, and interactions in biological samples, enabling in-depth exploration of disease-related proteomic signatures and functional networks [12]. For example, Chen et al. [13] used iTRAQ-PRM quantitative proteomics and found that Histone H2A, Histone H2B, and THBS1 were potential diagnostic biomarkers for chronic gout in patient plasma. Similarly, Lu et al. [14] applied 4D label-free proteomics to highlight the roles of complement and coagulation cascades, autophagy, lysosomal function, and purine metabolism in gouty arthritis. Metabolomics, particularly via non-targeted approaches, offers a comprehensive view of small-molecule metabolite diversity and abundance, allowing unbiased characterization of systemic metabolic alterations [15]. Through a non-targeted metabolomic analysis of urine from AGA and CGA patients, Jia et al. [16] identified 14 potential CGA biomarkers, such as acrylpyrazine, hypoxanthine, and xanthine, revealing associated dysregulation primarily in purine and amino acid metabolism. Wang et al. [17] further used metabolomics to uncover significant interplay disturbances between purine and caffeine metabolism in serum from patients with infrequent gout flares. Although proteomic and metabolomic studies have uncovered key biomarkers and dysregulated pathways in chronic gout, most research to date has relied on single-omics approaches. A significant gap still exists in the systematic elucidation of the coordinated proteomic and metabolomic regulatory networks that drive the dynamic transition from AGA to CGA. This lack of integrated multi-omics analysis hinders clarification of the dynamic crosstalk between pivotal proteins and core metabolic pathways, ultimately impeding the development of targeted therapies for chronic gout.

**Figure 1:**
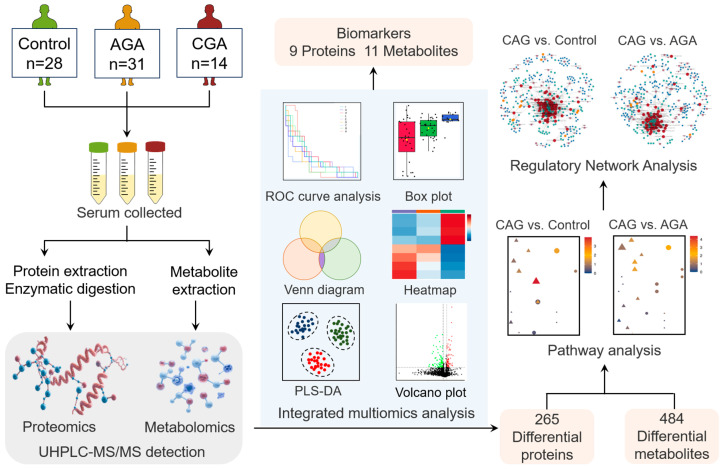
Research design and data analysis workflow. Control: healthy people; AGA: acute gouty arthritis; CAG: chronic gouty arthritis; UHPLC-MS/MS: ultrahigh-performance liquid chromatography–mass spectrometry; PLS-DA: partial least squares discriminant analysis; ROC: receiver operating characteristic.

In this study, we employ an innovative multi-omics integration strategy to explore and screen for high-potential candidate biomarkers for CAG diagnosis, aiming to elucidate the core molecular mechanisms underlying the progression from AGA to CGA. The research design and data analysis workflow is shown in Figure 1. We performed a cross-sectional analysis of serum samples from healthy controls (*n* = 28), AGA patients (*n* = 31), and CGA patients (*n* = 14) that integrated emerging high-depth data-independent acquisition (DIA) proteomics with non-targeted metabolomics. A differential analysis was then used to identify the dynamic molecular profiles and CGA-specific biomarkers. Through Kyoto Encyclopedia of Genes and Genomes (KEGG) pathway enrichment, Spearman correlation analysis, and Gene Ontology (GO) functional annotation, we constructed protein–metabolite interaction networks to uncover the core regulatory mechanisms of gout chronicity. This multi-omics integration provides a novel framework for understanding CGA progression and identifying potential therapeutic targets.

## 2. Materials and Methods

### 2.1. Reagents and Materials

The liquid chromatography–mass spectrometry (LC-MS) grade methanol, acetonitrile, and formic acid were obtained from Thermo Fisher Scientific (Waltham, MA, USA). The dithiothreitol, iodoacetamide, trifluoroacetic acid, ammonium bicarbonate, and triethylammonium bicarbonate buffer were purchased from Sigma-Aldrich (Shanghai, China). Urea was sourced from the Sinopharm Group (Shanghai, China). The MS-grade trypsin was acquired from Promega Biotech (Beijing, China). The Bradford protein quantification kit was supplied by Beyotime Biotechnology (Shanghai, China). The ProteoMiner™ kit was obtained from Bio-Rad Laboratories (Hercules, CA, USA) for low-abundance protein enrichment. The LC-MS grade water was procured from Thermo Fisher Scientific.

### 2.2. Blood Sample Collection

Peripheral venous blood samples were collected from 28 healthy controls, 31 AGA patients, and 14 CGA patients at the Rheumatology Department of the Air Force Medical Center using vacuum tubes. All participants fasted overnight prior to blood collection. Patient classification was confirmed by clinicians based on the diagnostic criteria. After a 30 min clotting period at room temperature, samples were centrifuged at 3500× *g* for 10 min. Serum aliquots were flash-frozen in liquid nitrogen and stored at −80 °C for the subsequent proteomic and metabolomic analyses. This research received approval from the Air Force Medical Center’s ethics committee and adhered to the principles of the 1964 Helsinki Declaration. Written informed consent was obtained from all participants.

### 2.3. Proteomic Analysis

Serum samples underwent high-abundance protein depletion using the Bio-Rad ProteoMiner™ enrichment kit (Bio-Rad Laboratories, Inc., Hercules, CA, USA) to enhance low-abundance species detections. Protein concentrations were subsequently determined using the Bradford assay kit (Bio-Rad, Hercules, CA, USA). Aliquots that contained 30 μg of protein were diluted in a dissolution buffer (8 M urea, 100 mM triethylammonium bicarbonate, pH 8.5) to a final volume of 100 μL. Trypsin was added with a 100 mM TEAB buffer, and digestion proceeded at 37 °C for 4 h. Additional trypsin was supplemented for overnight digestion. Reactions were terminated using 0.1% formic acid followed by centrifugation at 12,000× *g* for 5 min to remove precipitates. Supernatants were desalted using C18 solid-phase extraction columns. The eluate was lyophilized and reconstituted in 10 μL of 0.1% formic acid. After centrifugation at 14,000× *g* for 20 min, the supernatant was collected for the LC-MS analysis.

Chromatographic separation was performed using a Vanquish Neo ultra-high performance liquid chromatography (UHPLC) system coupled to an Orbitrap Astral mass spectrometer (Thermo Scientific, Waltham, MA, USA). Samples were loaded onto a PepMap C18 trap column (5 mm × 300 μm, 5 μm) and separated on a PepMap Neo ES906 analytical column (150 μm × 15 cm, 2 μm) (Thermo Scientific, Waltham, MA, USA) and maintained at 50 °C. The mobile phases consisted of (A) 0.1% formic acid in water and (B) 0.1% formic acid in 80% acetonitrile. A gradient elution program was employed with the following variable flow rates: initial conditions of 4% B at 2.5 μL/min transitioning to 8% B over 0.3 min while decreasing the flow to 1.5 μL/min; then, from 0.3 to 7.5 min, phase B increased to 22.5% at a constant flow (1.5 μL/min). This was followed by a rise to 35% B from 7.5 to 12.2 min. The gradient then progressed to 55% B over 0.4 min (12.2–12.6 min) while increasing the flow to 2.5 μL/min, followed by a rapid increase to 99% B over 0.4 min (12.6–13.0 min), maintained for 0.7 min (13.0–13.7 min) at 2.5 μL/min. The mass spectrometric analysis utilized data-independent acquisition (DIA) in positive ion mode. Full MS1 scans covered *m*/*z* 380–980 at a 240,000 resolution, while the DIA scans employed 300 variable isolation windows across *m*/*z* 150–2000 at an 80,000 resolution with higher-energy collisional dissociation (HCD) fragmentation.

### 2.4. Metabolomics Analysis

The freshly thawed serum aliquots (100 μL) were transferred to pre-chilled 1.5 mL microcentrifuge tubes. After the addition of 400 μL of ice-cold 80% methanol (*v*/*v*), the samples were vortex-mixed for 30 s and incubated on ice for 5 min to facilitate protein precipitation. Subsequent centrifugation at 15,000× *g* for 20 min at 4 °C was performed, followed by careful transfer of the supernatant to new microcentrifuge tubes. The supernatant was diluted with ultrapure water to achieve a final methanol concentration of 53% (*v*/*v*) and centrifuged again under identical conditions (15,000× *g*, 20 min, 4 °C). The resulting supernatant was collected for the ultra-high performance liquid chromatography coupled with tandem mass spectrometry (UHPLC-MS/MS) analysis.

The metabolomic analysis was performed using a Thermo Scientific Vanquish UHPLC system coupled to a Q Exactive HF mass spectrometer (Thermo Scientific, Waltham, MA, USA). Metabolites were separated on a Hypersil Gold column (100 × 2.1 mm, 1.9 μm) (Thermo Scientific, Waltham, MA, USA) maintained at 40 °C. The mobile phases consisted of (A) 0.1% formic acid in water and (B) methanol delivered at 0.2 mL/min. The gradient elution program proceeded as follows: 2% B for 1.5 min, increased to 85% B over 1.5 min, ramped to 100% B over 7 min, returned to 2% B in 0.1 min, and held at 2% B for 1.9 min. Mass spectrometry employed data-dependent acquisition in both the positive and negative ionization modes, with full MS scans spanning *m*/*z* 100–1500.

### 2.5. Data Processing and Statistical Analysis

The proteomic raw data were processed using DIA-NN 1.8.1 software with database searching against the UniProt human reference proteome. The search parameters included the following: 10 ppm precursor mass tolerance, 0.02 Da fragment mass tolerance, static carbamidomethyl modification of cysteine residues, and a maximum of two missed cleavages. To ensure the analytical quality, the results were filtered to retain only high-confidence peptides with a >99% confidence and the removal of peptide-spectrum matches and corresponding proteins that exceeded a 1% false discovery rate (FDR). The protein quantification values were normalized prior to the statistical analysis. The differential expression between groups was assessed using Student’s *t*-test, with significantly altered proteins identified based on a fold change (FC) threshold of >1.2 or <0.83 and a significance level of *p* < 0.05.

The metabolomics raw data were processed using Compound Discoverer 3.3 for peak alignment, feature extraction, and peak area quantification. The metabolite annotation was performed using multi-stage matching against the mzCloud (https://www.mzcloud.org/ (accessed on 12 June 2024)), mzVault, and MassList databases, utilizing the exact mass measurements (mass error < 5 ppm) of molecular ions and predicted fragment patterns. Quality control (QC)-based normalization (formula: sample peak area/[sum of sample metabolite quantifications/sum of QC1 metabolite quantifications]) was used to calculate the relative peak areas. To ensure data reliability, metabolites that exhibited >30% coefficients of variation in the QC samples were excluded prior to the downstream analysis. A multivariate statistical analysis was performed using the metaX platform that included a principal component analysis (PCA) and a partial least squares–discriminant analysis (PLS-DA). Differential metabolites were identified based on variable importance in the projection (VIP) scores >1.0 combined with a fold change (FC) threshold of >1.2 or <0.83 and statistical significance (*p* < 0.05) determined by Student’s *t*-test. Data visualization included volcano plots and clustered heatmaps. A KEGG pathway enrichment analysis of differential proteins and metabolites was conducted, and the results were visualized via bubble plots. A protein–metabolite interaction network was constructed using Spearman correlation analysis.

## 3. Results

### 3.1. Clinical Characteristics of the Selected Subjects

The clinical characteristics of the enrolled subjects are presented in Table 1. The serum uric acid levels were significantly elevated in both the AGA patients [median 481.0 and interquartile range (IQR) 336.0–696.0) μmol/L] and CGA patients [median 538.0 and IQR 388.2–718.0 μmol/L], compared to those of the healthy control group [median 342.0 and IQR 231.6–427.3 μmol/L]. Notably, the serum uric acid levels did not significantly differ between the CGA and AGA patient groups. Although participants with other metabolic disorders were excluded to minimize confounding, significant differences were observed between the CGA group and controls for age, smoking status, alcohol consumption, total cholesterol, and urea. Similarly, the AGA group significantly differed from the controls regarding age, body mass index (BMI), smoking status, alcohol consumption, sleep duration, alanine aminotransferase (ALT), glucose (GLU), and triglycerides (TG). However, with the exception of alcohol consumption, glucose, and blood urea nitrogen (BUN), there were no significant differences in these parameters between the CGA and AGA groups. Notably, although the mean BMI in the CGA group was significantly higher than in the controls, the majority of participants in this group were of normal weight or only mildly overweight.

### 3.2. Proteome Differential Expression Analysis

**Figure 2:**
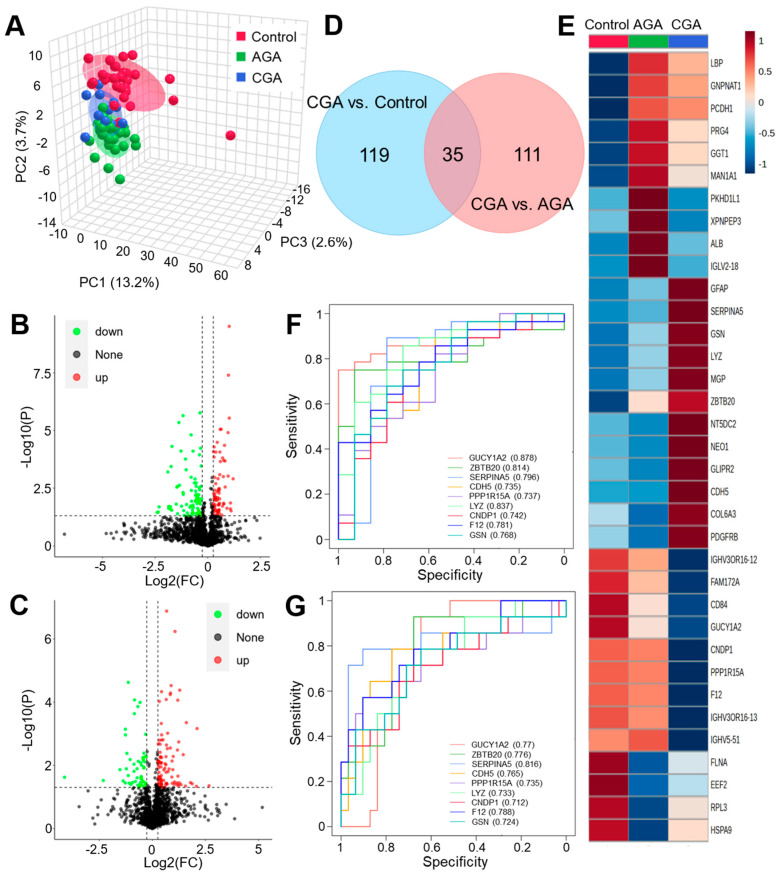
Differential proteomic analysis of CGA versus the control or AGA. (**A**) PCA analysis of proteomic profiles across the control, AGA, and CGA groups. PCA displays the distribution of all samples along the directions of maximum variance (PC1), the second greatest variance (PC2), and the third greatest variance (PC3). Percentages denote the variance explained by each component. Colors represent different experimental groups. Volcano plots that depict the dysregulated proteins in CGA versus (**B**) the control or (**C**) AGA. The x-axis shows the log2 fold change in protein expression, and the y-axis shows the −log10 *p*-value denoting statistical significance. Dashed lines mark significance thresholds (*p* < 0.05, |FC| > 1.2). Significantly upregulated (red points), downregulated (blue points), and non-significant (gray points) proteins are shown. (**D**) Venn diagram that identifies the unique and shared differentially expressed proteins. (**E**) Heatmap that visualizes the expression patterns of dysregulated proteins across the control, AGA, and CGA groups. ROC curve evaluation of the diagnostic potential of candidate biomarkers for CGA versus (**F**) the control or (**G**) AGA.

We performed a DIA-based quantitative proteomic analysis on 73 serum samples that encompassed the healthy controls, AGA, and CGA patients. Our systematic quality control of the proteomic data showed that the peptide length distribution (7–30 amino acids) reflected robust and reproducible digestion (Figure S1A). The high consistency in the indexed retention time (iRT) of the internal standard-calibrated peptides attested to stable MS performance, while the abundance of unique peptides and their flat cumulative distribution confirmed the reproducibility of protein identification (Figure S1B,C). A total of 2548 proteins were identified and quantified. The PCA revealed distinct proteomic profiles among the control, AGA, and CGA groups within the 95% confidence region. While the control and AGA groups exhibited clear separation, the CGA group showed partial overlap with both the control and AGA profiles (Figure 2A). To identify differentially expressed proteins during CGA progression, volcano plots were generated using thresholds of |FC| > 1.2 (FC < 0.83 or FC > 1.2) and statistical significance (*p* < 0.05) (Figure 2B,C). This analysis identified 154 significantly dysregulated proteins in CGA vs. control comparisons (73 upregulated, 82 downregulated) and 146 in the CGA vs. AGA comparisons (54 upregulated, 92 downregulated).

The GO functional enrichment analysis revealed that differentially expressed proteins (DEPs) in the CGA vs. control comparison were significantly enriched in 19 GO terms (*p* < 0.05), primarily within the molecular function categories (Figure S2). In contrast, only three GO terms were significantly enriched for DEPs in the CGA vs. AGA comparison (*p* < 0.05). This result indicated higher similarity in the biological functions between the CGA and AGA serum proteomes compared to the healthy controls. The Venn diagram analysis identified 35 persistently dysregulated proteins throughout CGA progression. Additionally, 119 DEPs were unique to CGA vs. control, while 111 DEPs were specific to CGA vs. AGA (Figure 2D). The hierarchical clustering heatmap visualization demonstrated expression patterns of these 35 shared proteins during CGA progression (Figure 2E). A total of 12 DEPs were further selected from this common pool based on consistent directional changes observed in the boxplot analyses (Figure S3). The ROC curve evaluation showed that nine of these core proteins exhibited area under the curve (AUC) values of >0.7 to discriminate CGA from the control or AGA (Figure 2F,G, Table 2). Subsequent five-fold cross validation further confirmed the robust performance of these proteins in both the discovery and validation sets, with a mean AUC significantly greater than 0.65 (Figure S4). This result suggested their potential as CGA diagnostic or progression-monitoring biomarkers.

### 3.3. Metabolome Difference Analysis

**Figure 3:**
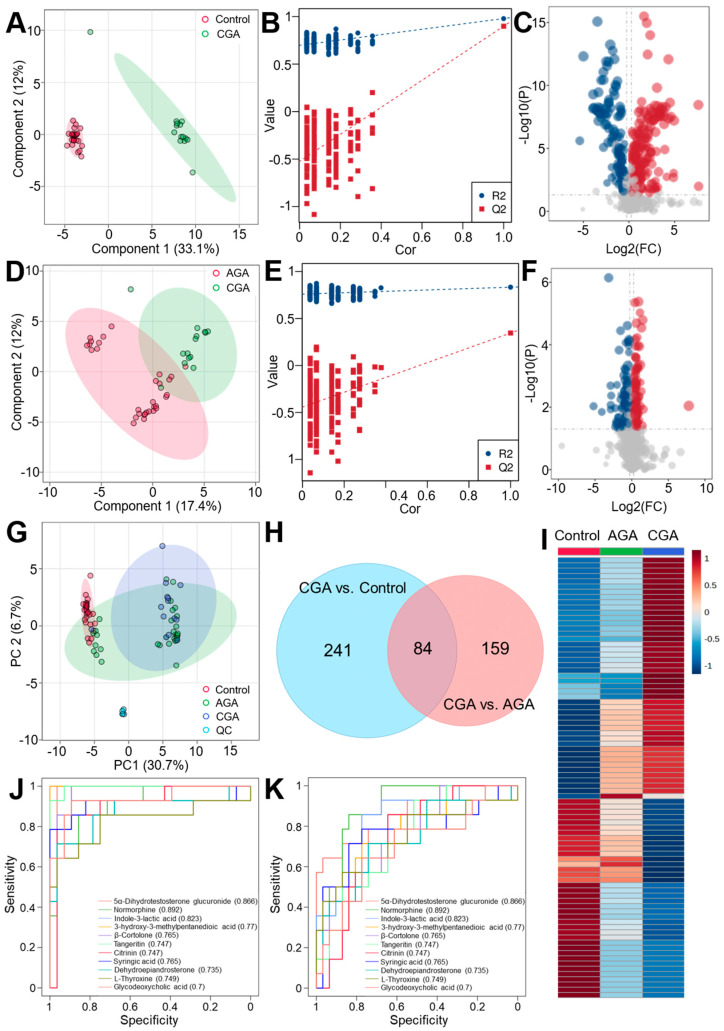
Differential metabolomic analysis of CGA versus the control or AGA. (**A**–**C**) CGA vs. control comparisons: (**A**) PLS-DA score plot, (**B**) permutation validation, and (**C**) volcano plot of the differentially abundant metabolites. (**D**–**F**) CGA vs. AGA comparisons: (**D**) PLS-DA score plot, (**E**) permutation validation, and (**F**) volcano plot. PLS-DA score plot showing sample distribution along the first two latent components (Component 1 and Component 2), capturing the major variation that separates the predefined groups. Colors denote the different experimental groups. Volcano plot of differential metabolites. The x-axis represents the log_2_ fold change in metabolite abundance, and the y-axis represents the −log_10_ *p*-value. Point size corresponds to the variable importance in projection score from the PLS-DA model. Dashed lines indicate significance thresholds (VIP > 1, *p* < 0.05, |FC| > 1.2). Red, blue, and grey points denote significantly upregulated, downregulated, and non-significant metabolites, respectively. (**G**) PCA analysis of global metabolomic profiles. (**H**) Venn diagram analysis of the unique and shared differential metabolites. (**I**) Heatmap visualization analysis of the metabolite abundance patterns. ROC curves for the candidate metabolites that discriminated CGA from (**J**) the control or (**K**) AGA.

UPLC-MS/MS in both positive and negative ion modes was used to perform the serum metabolomic profiling of the control, AGA, and CGA groups. Quality control samples were integrated throughout the analytical process to ensure data reliability. The PCA demonstrated tight clustering of the QC samples within the 95% confidence region that confirmed instrument stability and analytical reproducibility (Figure 3G). The control and CGA samples showed complete separation in the PCA score plot, while both partially overlapped with the AGA samples. This pattern suggested a metabolic continuum from healthy states to AGA and ultimately CGA. The PLS-DA models were subsequently constructed to enhance the group discrimination (Figure 3A,D). Permutation tests confirmed the model validity (Figure 3B,E), with R2 values exceeding Q2 and negative Y-axis intercepts for the Q2 regression lines. This result indicated the absence of overfitting and a strong predictive capability. The PLS-DA results corroborated the PCA findings, revealing a substantial metabolic divergence between the CGA and control groups, with intermediate differences between the CGA and AGA cohorts.

The differential metabolites were identified in the CGA vs. control (n = 325) and CGA vs. AGA (n = 243) comparisons using thresholds of VIP of >1.0 from PLS-DA (95% confidence region), *p* < 0.05 from *t*-test, and |FC| of >1.2 (FC < 0.83 or FC > 1.2). Specifically, 198 metabolites were upregulated and 127 downregulated in the CGA vs. control (Figure 3C), versus 139 upregulated and 104 downregulated in the CGA vs. AGA (Figure 3F). This demonstrated substantially higher metabolic disparity between CGA and the control than between CGA and AGA, suggesting sustained metabolic reprogramming during progression. The Venn analysis revealed 84 metabolites that were dysregulated in both comparisons (Figure 3H), with 241 unique to CGA vs. control and 159 to CGA vs. AGA. The hierarchical clustering heatmap of these 84 shared metabolites showed distinct abundance patterns across the groups (Figure 3I). The boxplot analysis identified 18 metabolites with consistent directional changes among the control, AGA, and CGA (Figure S4), all annotatable in the human metabolome database (HMDB), KEGG, and LipidMaps. The ROC evaluation further refined 11 core metabolites with AUCs of >0.7 to discriminate CGA from the control or AGA (Figure 3J,K, Table 3). Further five-fold cross validation demonstrated that the mean AUC of these metabolites was significantly higher than 0.7 in both the discovery and validation sets (Figure S5). Notably, these metabolites showed superior discriminations for CGA vs. control compared to CGA vs. AGA. Collectively, these 11 metabolites demonstrated considerable diagnostic potential for CGA.

### 3.4. Pathway Analysis

**Figure 4:**
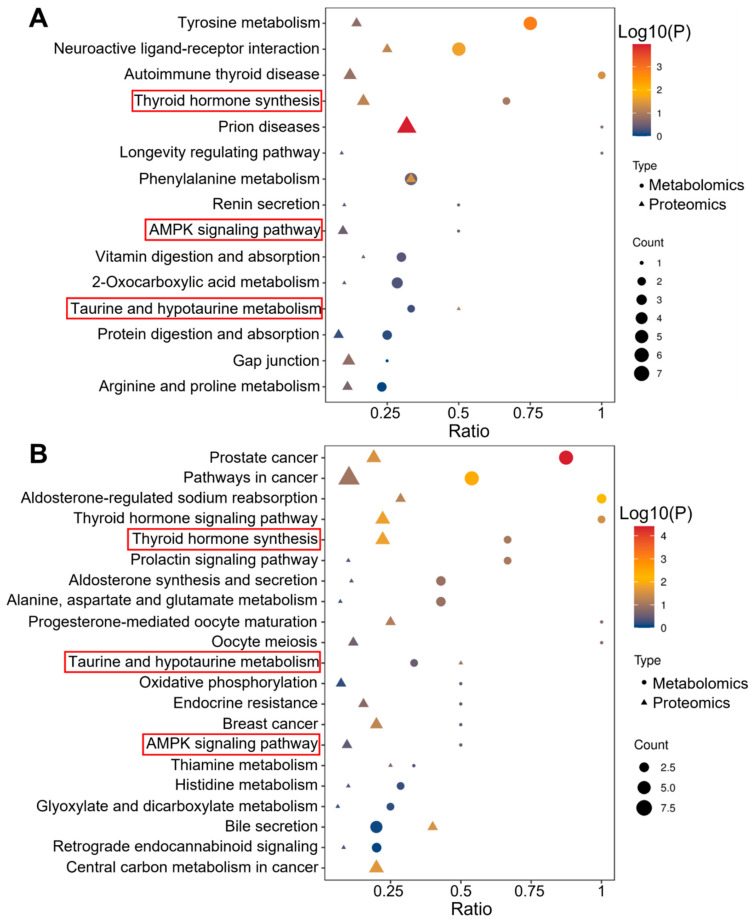
Functional characterization of the dysregulated proteins and metabolites in the CGA group compared to the (**A**) control or (**B**) AGA groups. The pathways circled in red box are the pathways where differential proteins and metabolites are co-enriched.

The multi-omics pathway analysis mapped differentially expressed proteins and metabolites from CGA vs. control and the CGA vs. AGA comparisons to the KEGG pathways. The CGA vs. control analysis identified 15 significantly enriched pathways (Figure 4A, Table S1. The prion diseases (*p* = 1.11 × 10^−4^) and phenylalanine metabolism (*p* = 3.97 × 10^−2^) demonstrated protein-level enrichment, while the tyrosine metabolism (*p* = 1.47 × 10^−3^), neuroactive ligand-receptor interactions (*p* = 2.34 × 10^−2^), and autoimmune thyroid disease (*p* = 4.48 × 10^−2^) showed metabolite-level enrichment. In contrast, the CGA vs. AGA comparison revealed enrichment across 21 pathways (Figure 4B, Table S2), including prostate cancer (protein *p* = 2.92 × 10^−2^; metabolite *p* = 3.97 × 10^−5^) and thyroid hormone signaling (protein *p* = 1.71 × 10^−2^; metabolite *p* = 3.47 × 10^−2^), which exhibited dysregulation at both molecular levels. An additional protein-level dysregulation was observed in aldosterone-regulated sodium reabsorption (*p* = 6.31 × 10^−3^), thyroid hormone synthesis (*p* = 1.71 × 10^−2^), central carbon metabolism in cancer (*p* = 2.47 × 10^−2^), and bile secretion (*p* = 2.95 × 10^−2^). Collectively, pathway alterations demonstrated greater complexity in CGA vs. AGA than in the CGA vs. control comparisons.

**Figure 5:**
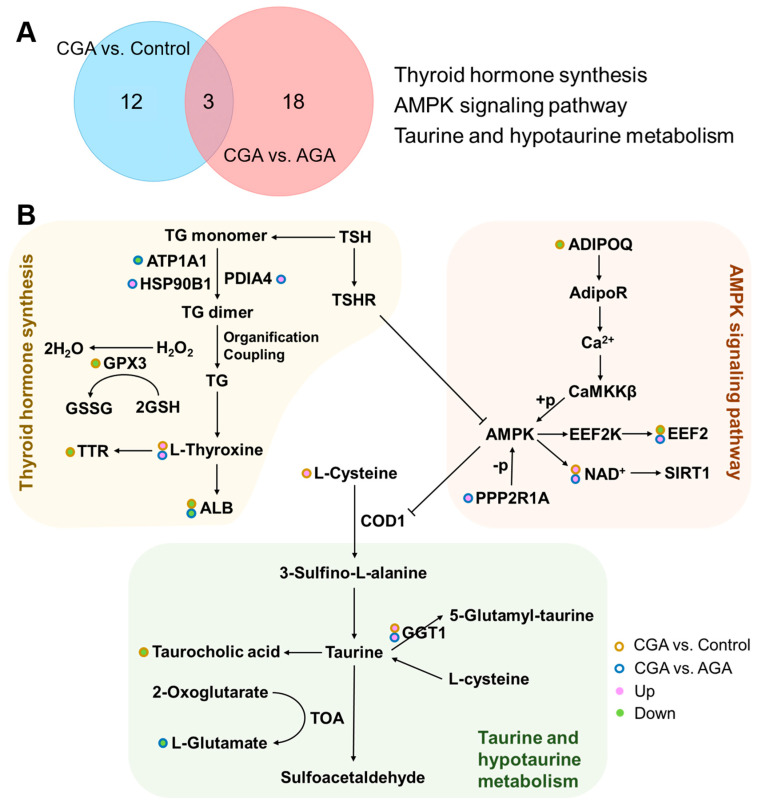
Pathway analysis. (**A**) Venn diagram that identifies the number of pathways co-enriched by significantly altered proteins and metabolites in both the CGA vs. control and CGA vs. AGA comparisons. Pathways commonly regulated in both comparative groups are listed. (**B**) Protein and metabolite regulatory alterations within the core pathways: thyroid hormone synthesis, AMPK signaling pathway, and taurine and hypotaurine metabolism.

To investigate the regulation of biological pathways during the progression from AGA to CGA in gout patients, Venn diagram analysis was performed to identify pathways co-enriched in both the CGA vs. control and CGA vs. AGA comparisons (Figure 5A). Three pathways were co-enriched: thyroid hormone synthesis (related to thyroid function), the AMPK signaling pathway (a core regulator of cellular energy metabolism), and taurine and hypotaurine metabolism (a key branch of sulfur-containing amino acid metabolism). This co-enrichment suggested that these pathways may be coregulated during CGA pathogenesis. Figure S6 quantifies the differentially expressed proteins and metabolites within these co-enriched pathways. The thyroid hormone synthesis pathway included L-thyroxine, ATP1A1, HSP90B1, PDIA4, GPX3, TTR, and ALB. The AMPK signaling pathway featured NAD+, ADIPOQ, EEF2, and PPP2R1A. The taurine and hypotaurine metabolism pathway encompassed taurocholic acid, L-cysteine, L-glutamate, and GGT1. Figure 5B illustrates the protein and metabolite alterations associated with these core pathways, demonstrating that several were commonly regulated in both comparisons. Our data also revealed specific differences between the two comparison groups.

### 3.5. Regulatory Network Analysis

**Figure 6:**
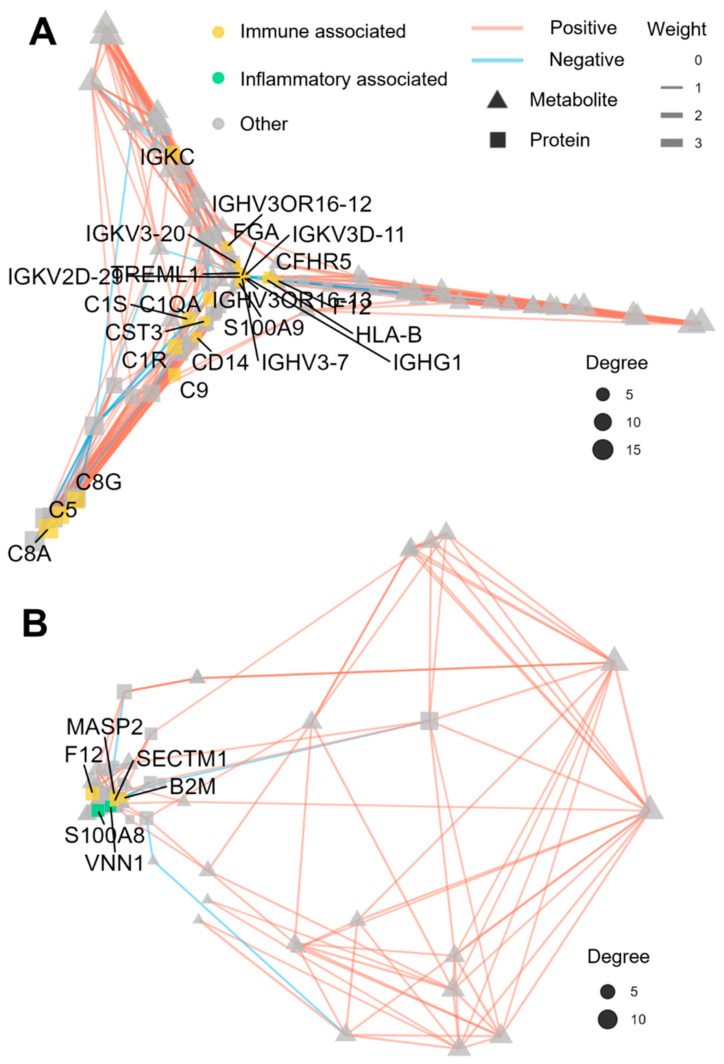
Regulatory network analyses of the biologically functional proteins (squares) and metabolites (triangles) in (**A**) CGA vs. control and (**B**) CGA vs. AGA comparisons.

A paired correlation network analysis was performed on all of the differentially expressed proteins and metabolites to identify the co-regulated nodes. Figure 6 highlights the most highly correlated proteins and metabolites within the CGA group. In this network, the nodes represent the differentially expressed proteins (squares) or metabolites (triangles). The node size is proportional to its degree, reflecting the relative importance within the network. Connections (edges) between the nodes were retained, where the absolute Spearman correlation coefficient was ≥0.7. Positive and negative correlations are denoted by the red and blue edges, respectively. The edge width is proportional to the absolute correlation coefficient, while the node distance is inversely proportional to it. The protein nodes were annotated based on the GO biological process enrichment analysis to facilitate interpretation.

For the CGA vs. control comparison, the constructed network comprised 156 nodes that included 67 differentially expressed proteins (squares) and 89 metabolites (triangles). Among these, 23 protein nodes (yellow-highlighted) were closely associated with immune function, suggesting significant immunomodulation during CGA formation. These proteins were enriched in immune-related biological processes that included complement activation (classical, alternative), innate immune response, adaptive immune response, immunoglobulin-mediated immune response, and positive regulation of immune response. Figure 6A highlights the key regulatory proteins within these immune pathways. For the CGA vs. AGA comparison, the network contained 133 nodes that included 51 differentially expressed proteins (squares) and 82 metabolites (circles). Only four protein nodes (yellow-highlighted) demonstrated strong immune associations, enriched specifically in positive regulation of T cell cytokine production and the innate immune response. Additionally, two protein nodes (green-highlighted) were linked to inflammatory processes enriched in the chronic inflammatory response. Figure 6B depicts the key protein nodes within these immune and inflammatory pathways. Notably, the network analysis revealed a higher emphasis on the chronic inflammatory responses in CGA vs. AGA compared to CGA vs. the control (Figure S7).

## 4. Discussion

The AGA to CGA transition represents a central pathological process that drives irreversible joint damage and systemic complications [18,19,20,21]. Previous mechanistic studies have predominantly relied on single-omics approaches, limiting a comprehensive understanding of the multi-omics dynamics underlying this transition [11,13,14,16,17]. To address this gap, we integrated high-depth DIA proteomics with untargeted metabolomics to analyze serum samples from healthy controls, AGA, and CGA patients. Our findings provide a systematic multi-omics perspective on the AGA-to-CGA progression, identifying persistently dysregulated molecular clusters and their associated regulatory networks.

Our proteomic analysis identified nine pivotal proteins that were consistently dysregulated and demonstrated strong diagnostic potential (AUC > 0.7) in both CGA vs. control and CGA vs. AGA comparisons. Among these, the upregulation of ZBTB20, a transcriptional repressor of the urate transporter ABCG2, may explain the persistent hyperuricemia observed in CGA by impairing renal urate excretion. This observation aligns with prior studies that have associated ABCG2 dysfunction with increased gout severity [22]. Concurrently, the downregulation of GUCY1A2, a key enzyme in cyclic guanosine monophosphate (cGMP) synthesis, suggests diminished anti-inflammatory signaling, potentially exacerbating inflammation via NF-κB activation [23]. These findings are consistent with studies highlighting the role of cGMP in modulating inflammatory responses in chronic inflammatory diseases.

We also observed alterations in proteins involved in immune and coagulation pathways. Reduced levels of CNDP1, an anti-inflammatory enzyme [24], along with elevated levels of the antibacterial protein LYZ [25,26], indicate persistent immune dysregulation. Similarly, increased anticoagulant regulator SERPINA5 [27,28] and decreased coagulation factor F12 [29] suggest a shift in coagulation dynamics, which may contribute to the pro-thrombotic state reported in gout patients. The dysregulation of GSN, an actin-regulatory protein, further supports the notion of impaired immunomodulation and tissue repair in CGA [30]. Elevated CDH5 levels in both AGA and CGA sera point to vascular endothelial injury as an early and sustained event in gout progression [31,32], while decreased PPP1R15A implies compromised cellular stress responses [33,34]. Collectively, these proteomic alterations underscore the interplay between hyperuricemia, inflammation, coagulation, and endothelial dysfunction in CGA pathogenesis.

Metabolomic profiling revealed eleven core metabolites that were consistently altered in CGA. The reduction in 5α-dihydrotestosterone glucuronide and increase in dehydroepiandrosterone support the involvement of sex hormone imbalances in gout, as previously suggested [35]. The accumulation of gut microbiota-derived metabolites, such as indole-3-lactic acid and 3-hydroxy-3-methylpentanedioic acid, along with decreased glycodeoxycholic acid, indicates gut dysbiosis and systemic translocation of microbial products. This phenomenon is increasingly implicated as a driver of chronic inflammation [36]. The presence of citrinin, a mycotoxin, hints at environmental exposures potentially exacerbating gout progression. Notably, the detection of normorphine and exogenous L-thyroxine may reflect pharmaceutical exposure or metabolic abnormalities [37], while altered levels of dietary metabolites like syringic acid and tangeritin suggest nutritional influences on CGA risk. The elevation of β-cortolone, a cortisol metabolite, implies relative cortisol deficiency or increased consumption, possibly compromising endogenous anti-inflammatory capacity [38]. These metabolomic findings highlight a complex network of exogenous, endogenous, and nutritional factors contributing to CGA.

Integrative pathway analysis identified thyroid hormone synthesis, AMPK signaling, and taurine and hypotaurine metabolism as central pathways dysregulated during the AGA-to-CGA transition. The coordinated alterations in L-thyroxine, ATP1A1, and TTR within the thyroid hormone synthesis pathway suggest hypothalamic–pituitary–thyroid axis involvement, which may influence metabolic and inflammatory processes in gout [37,39]. Although this inference is supported by the existing literature, further experimental validation is needed. Activation of the NAD+-ADIPOQ-AMPK axis and disrupted taurine metabolism were associated with impaired tissue repair [40] and oxidative stress [41], respectively, highlighting metabolic reprogramming as a key feature of CGA.

Correlation network analysis further revealed immune microenvironment remodeling during CGA development. While CGA vs. control comparisons showed broad immunoregulatory imbalances, CGA vs. AGA analyses highlighted chronic inflammatory responses, indicating a shift from acute immune activation to sustained inflammation. This immunophenotypic evolution aligns with the clinical progression of gout and suggests potential targets for immunomodulatory interventions.

This multi-omics study advances beyond previous single-omics reports by integrating proteomic and metabolomic data to capture the complexity of gout chronicization. For instance, while Chen et al. [13] and Lu et al. [14] identified protein markers and pathways in chronic gout using targeted proteomic approaches, our study combined DIA proteomics with untargeted metabolomics to reveal a broader molecular landscape. Similarly, compared to urine metabolomic studies by Jia et al. [16] and Wang et al. [17], our serum-based analysis detected a wider array of metabolites and integrated them with protein data to construct dynamic interaction networks. Furthermore, the integrated pathway enrichment analysis pointed to a novel pathway network in which the thyroid hormone–AMPK–taurine metabolism axis might represent a key cooperative mechanism underlying CGA pathogenesis, offering new avenues for multi-target therapeutic strategies.

Although this study has made remarkable progress, there remain several limitations. First, the CGA cohort had a relatively small sample size and lacked an independent external cohort for validating the candidate biomarkers. Future studies should expand the sample size and validate these candidates in prospective and independent cohorts. Second, the cross-sectional design inherently precludes causal inference regarding the identified biomarkers and pathways. Longitudinal studies with functional validation (in vitro/in vivo) are needed to establish causality. Third, the core molecular clusters and pathways require experimental verification. Future investigations should employ independent biological replicates to verify key targets at the protein level via western blot or ELISA. The functional roles of these candidates and pathways in MSU crystal-induced inflammation and cartilage degradation should be investigated using genetic manipulation or pharmacological interventions in monocytes, synoviocytes, macrophages, chondrocytes, and urate oxidase knockout mouse models. Finally, the lack of detailed clinical dietary intake data in this study precluded an assessment of the potential contribution of exogenous dietary metabolites to CGA onset. Future research should integrate dietary questionnaires or systematic nutritional records to further explore the possible mechanisms by which diet-derived metabolites influence the development of CGA.

## 5. Conclusions

In this study, we integrated proteomic and metabolomic analyses to systematically identify persistently dysregulated molecular clusters during the progression from AGA to CGA and evaluate their potential diagnostic biomarker values. We further elucidated the core regulatory role of a novel thyroid hormone–AMPK–taurine metabolic axis that contributes to gout chronicity and characterized the transition of immune signatures from acute activation to chronic inflammation. Despite inherent study design and technical limitations, the key molecular clusters and dysregulated pathways identified in this study provide a crucial foundation for the clinical development of early-warning CGA biomarkers and targeted therapeutic strategies. Future longitudinal multi-omics studies combined with mechanistic experimentation and clinical validation may facilitate the translation of these findings into effective interventions to improve long-term outcomes for gout patients.

## Figures and Tables

**Table 1:** Clinical and demographic characteristics of the study participants *.

	Normal Controls (n = 28)	Patients with AGA (n = 31)	Participants with CGA (n = 14)
Age, years, median (IQR)	26 (24–28)	33 (22–51) ^†^	28 (21–54) ^†^
BMI, kg/m^2^, median (IQR)	23.2 (19.6–28.4)	26.5 (17.0–32.0) ^†^	24.8 (19.6–34.3)
Smoking, n(%) ^a^	2 (10.5)	20 (66.7) ^†^	9 (64.3) ^†^
Drinking, n(%) ^b^	0 (0)	11 (36.7) ^†^	2 (14.3)
Beverage, n(%) ^c^	8 (42.1)	13 (43.3)	1 (7.1) ^†,‡^
Sleep time, hours/day, median (IQR)	7 (6–8)	7 (4–8) ^†^	7 (6–9)
ALT, units/liter, median (IQR)	25.1 (7.7–70.8)	42.4 (16.9–91.6) ^†^	30.7 (11.1–92.5)
AST, units/liter, median (IQR)	20.6 (8.9–67.6)	25.5 (10.4–54)	21.9 (15.4–41.5)
GLU, mmoles/liter, median (IQR)	4.7 (3.5–5.6)	5.0 (3.8–6.8) ^†^	4.7 (4.1–5.6) ^‡^
TG, mmoles/liter, median (IQR)	0.9 (0.5–2.2)	1.4 (0.7–4.2) ^†^	1.3 (0.7–1.5)
TCH, mmoles/liter, median (IQR)	4.7 (3.4–6.1)	4.6 (1.8–6.9)	3.9 (3.1–4.9) ^†^
BUN, mmoles/liter, median (IQR)	5.5 (3.7–8.4)	5.1 (3.9–7.7)	4.3 (3.0–7.0) ^†,‡^
CR, mmoles/liter, median (IQR)	78.5 (62.0–93.0)	82.1 (65.0–106.0)	93.0 (60.0–112.0)
SUA, µmoles/liter, median (IQR)	342.0 (231.6–427.3)	481.0 (336.0–696.0) ^†^	538.0 (388.2–718.0) ^†^

* Complete data were not available for every participant. Values for continuous variables are presented as medians (quartile). Values for categorical variable data are presented as n (%). BMI = body mass index; ALT = alanine aminotransferase; AST = aspartate aminotransferase; GLU = glucose; TG = triglycerides; TCH = total cholesterol; BUN = blood urea nitrogen; CR = creatinine; SUA = serum urate acid. ^a^ At least 20 cigarette packs in a lifetime or at least one cigarette a day for at least one year. ^b^ Alcohol intake at least once a week for six months. ^c^ Beverage intake at least twice a week for six months. ^†^ *p* < 0.05 versus normal controls. ^‡^ *p* < 0.05 versus participants with AGA.

**Table 2:** Shared differentially expressed proteins with consistent dysregulation and diagnostic utility in CGA versus the control or AGA.

No.	Protein	CGA vs. Control				CGA vs. AGA				Up. Down
		*p*	FC	log2FC	AUC	*p*	FC	log2FC	AUC	
1	GUCY1A2	4.52 × 10^−6^	0.39	−1.37	0.878	2.37 × 10^−5^	0.46	−1.13	0.77	down
2	ZBTB20	2.10 × 10^−4^	1.77	0.82	0.814	1.55 × 10^−3^	1.41	0.50	0.776	up
3	SERPINA5	1.99 × 10^−4^	1.69	0.76	0.796	2.96 × 10^−3^	1.63	0.70	0.816	up
4	CDH5	1.06 × 10^−2^	1.28	0.35	0.735	5.65 × 10^−3^	1.29	0.37	0.765	up
5	PPP1R15A	7.01 × 10^−3^	0.72	−0.48	0.737	6.88 × 10^−3^	0.74	−0.43	0.735	down
6	LYZ	8.69 × 10^−5^	1.30	0.38	0.837	7.33 × 10^−3^	1.20	0.27	0.733	up
7	CNDP1	8.64 × 10^−3^	0.73	−0.45	0.742	1.14 × 10^−2^	0.76	−0.40	0.712	down
8	F12	9.37 × 10^−3^	0.73	−0.46	0.781	1.21 × 10^−2^	0.76	−0.39	0.788	down
9	GSN	1.45 × 10^−2^	1.48	0.56	0.768	4.07 × 10^−2^	1.35	0.44	0.724	up

**Table 3:** Annotated shared metabolites with consistent dysregulation and diagnostic utility in CGA vs. control and CGA vs. AGA comparisons.

No.	Metabolite	CGA vs. Control						CGA vs. AGA				
		FC	log2FC	*p*	VIP	AUC	FC	log2FC	*p*	VIP	AUC	Up. Down
1	5α-Dihydrotestosterone glucuronide	0.33	−1.60	8.24 × 10^−13^	2.31	0.982	0.69	−0.54	8.13 × 10^−5^	1.09	0.866	down
2	Normorphine	0.39	−1.37	2.97 × 10^−4^	1.16	0.911	0.55	−0.86	8.60 × 10^−5^	1.13	0.892	down
3	Indole-3-lactic acid	5.46	2.45	1.28 × 10^−8^	2.51	1	1.90	0.93	5.79 × 10^−4^	1.60	0.823	up
4	3-hydroxy-3-methylpentanedioic acid	20.06	4.33	8.46 × 10^−13^	2.32	1	2.38	1.25	1.61 × 10^−3^	1.04	0.77	up
5	β-Cortolone	4.67	2.22	9.77 × 10^−6^	2.05	0.982	2.46	1.30	3.11 × 10^−3^	1.74	0.765	up
6	Tangeritin	0.56	−0.84	1.56 × 10^−8^	1.94	0.995	0.65	−0.61	3.34 × 10^−3^	1.48	0.747	down
7	Citrinin	1.51	0.60	4.61 × 10^−5^	1.47	0.906	1.32	0.40	3.92 × 10^−3^	1.48	0.747	up
8	Syringic acid	3.44	1.78	1.38 × 10^−4^	2.16	0.913	1.96	0.97	4.53 × 10^−3^	2.09	0.765	up
9	Dehydroepiandrosterone	2.35	1.23	3.89 × 10^−4^	1.73	0.872	1.64	0.71	1.90 × 10^−2^	1.53	0.735	up
10	L-Thyroxine	1.39	0.47	7.26 × 10^−3^	1.21	0.827	1.28	0.35	3.34 × 10^−2^	1.38	0.749	up
11	Glycodeoxycholic acid	0.21	−2.28	3.47 × 10^−6^	2.01	0.934	0.48	−1.06	3.51 × 10^−2^	1.22	0.7	down

## Data Availability

The original contributions presented in this study are included in the article/Supplementary Materials. Further inquiries can be directed to the corresponding authors.

## Supplementary Materials

The following supporting information can be downloaded at https://www.mdpi.com/article/10.3390/cells15010041/s1, Figure S1: Quality control analysis of DIA-based quantitative proteomic data. (A) Peptide length range distribution plot. (B) iRT values of internal standard calibrated peptides. A-L represent the internal standard calibrated peptides. (C) Distribution plot of unique peptide counts in identified proteins; Figure S2: Functional enrichment analysis of GO with statistical significance for differential proteins in (A) CGA vs. control and (B) CGA vs. AGA groups; Figure S3: Box plot analysis of differential proteins exhibiting consistent expression trends across control, AGA, and CGA groups; Figure S4: Results of the five-fold cross validation for the core proteins in the CAG vs. control and CAG vs. AGA comparisons across the discovery and validation sets; Figure S5: Results of the five-fold cross validation for the core metabolites in the CAG vs. control and CAG vs. AGA comparisons across the discovery and validation sets; Figure S6: Box plot analysis of differential metabolites exhibiting consistent trends across control, AGA, and CGA groups with annotations in HMDB, KEGG, and LipidMaps databases; Figure S7: Quantification of differentially expressed proteins and metabolites in pathways commonly enriched in (A) CGA vs. control and (B) CGA vs. AGA comparisons; Table S1 KEGG pathway enrichment analysis of differentially expressed proteins and differential metabolites in CGA vs. control comparison. Table S2 KEGG pathway enrichment analysis of differentially expressed proteins and differential metabolites in CGA vs. AGA comparison.

## Abbreviations

The following abbreviations are used in this manuscript:

AGA	acute gouty arthritis
CGA	chronic gouty arthritis
DIA	data-independent acquisition
KEGG	Kyoto Encyclopedia of Genes and Genomes
GO	Gene Ontology
LC-MS	liquid chromatography–mass spectrometry
UHPLC	ultra-high performance liquid chromatography
HCD	higher-energy collisional dissociation
UHPLC-MS/MS	ultra-high performance liquid chromatography coupled with tandem mass spectrometry
QC	quality control
PCA	principal component analysis
PLS-DA	partial least squares–discriminant analysis
VIP	variable importance in the projection
FC	fold change
IQR	interquartile range
BMI	body mass index
ALT	alanine aminotransferase
GLU	glucose
TG	triglycerides
BUN	blood urea nitrogen
DEPs	differentially expressed proteins
AUC	area under the curve
