# Supplementary material for: Multi-Omics Mechanism of Chronic Gout Arthritis and Discovery of the Thyroid Hormone–AMPK–Taurine Metabolic Axis
Source: Cells. 2025 Dec 25;15(1):41. doi: 10.3390/cells15010041 (PMC12785424; doi:10.3390/cells15010041)
Supplement: Supplementary file 1 [file cells-15-00041-s001.zip › Differential metabolites in CGA vs AGA comparison.pdf]

Differential metabolites in CGA vs AGA comparison

No.	Name	FC	log2FC	Pvalue	VIP	Up.Down
1	Cer 30:0;2O/12:0;O(FA 18:1)	0.11	-3.14	7.18E-07	**No.**	down
2	Etiocholanolone	1.90	0.93	4.18E-06	2.40	up
3	2-Phenylacetamide	1.35	0.44	4.48E-06	2.09	up
4	Bisphenol G	2.18	1.13	9.37E-06	1.58	up
5	LPC O-22:2	2.82	1.49	1.62E-05	2.34	up
6	Testosterone	1.56	0.64	2.03E-05	2.09	up
7	2-methyl-1,2-dihydrophthalazin-1-one	0.66	-0.59	2.44E-05	1.98	down
8	LPC O-20:1	2.52	1.33	2.87E-05	2.27	up
9	PE 34:2	0.35	-1.52	3.56E-05	1.74	down
10	PC O-36:3	1.67	0.74	5.86E-05	1.73	up
11	Nervonic acid	1.43	0.51	6.62E-05	1.73	up
12	PC O-39:4	0.76	-0.39	6.85E-05	2.02	down
13	Sulfamerazine	0.81	-0.30	7.65E-05	2.29	down
14	5α-Dihydrotestosterone glucuronide	0.69	-0.54	8.13E-05	1.09	down
15	Normorphine	0.55	-0.86	8.60E-05	1.13	down
16	4-Hydroxytamoxifen	0.75	-0.42	1.07E-04	1.31	down
17	3-Acetoxyurs-12-en-23-oic acid	1.87	0.90	1.09E-04	1.68	up
18	Cortisone	2.02	1.02	1.13E-04	2.38	up
19	gamma-Tocopherol	1.46	0.54	1.13E-04	1.66	up
20	PE 20:3_20:3	0.30	-1.72	1.49E-04	1.80	down
21	3-(3-morpholinopropyl)-2-(2-pyridinyl)-2,3-dihydro-4(1H)-quinazolinone	0.77	-0.38	1.75E-04	1.23	down
22	5-Hydroxytryptophan	1.40	0.48	2.40E-04	1.60	up
23	T-2 Toxin	1.47	0.56	2.53E-04	2.10	up
24	Levothyroxine	1.51	0.59	2.63E-04	1.77	up
25	2-(2-oxo-2-{[2-(2-oxo-1-imidazolidinyl)ethyl]amino}ethoxy)acetic acid	1.84	0.88	4.40E-04	1.66	up
26	Corey Lactone Diol	2.20	1.14	4.42E-04	2.71	up
27	(2S)-2-(2-hydroxypropan-2-yl)-2H,3H,7H-furo[3,2-g]chromen-7-one	0.70	-0.51	4.70E-04	2.67	down
28	LPC O-18:0	1.67	0.74	5.27E-04	1.94	up
29	PC 20:3_20:3	0.51	-0.97	5.78E-04	1.53	down
30	Indole-3-lactic acid	1.90	0.93	5.79E-04	1.60	up
31	Pregnenolone	1.65	0.73	5.85E-04	1.91	up
32	LPE 22:4	1.45	0.53	6.40E-04	1.64	up
33	Glycyl-L-leucine	0.56	-0.83	6.52E-04	1.77	down
34	N-[2-chloro-6-(trifluoromethoxy)phenyl]-2,2-dimethylpropanamide	1.40	0.48	7.27E-04	2.06	up
35	LPC 22:4-SN1	1.44	0.52	9.09E-04	1.94	up
36	2-oxa-4-azatetracyclo[6.3.1.1~6,10~.0~1,5~]tridecan-3-one	0.48	-1.05	9.61E-04	2.01	down

37	(2R)-5-hydroxy-7-methoxy-2-phenyl-3,4-dihydro-2H-1-benzopyran-4-one	0.65	-0.63	1.00E-03	1.66	down
38	5α-Dihydrotestosterone	1.27	0.35	1.06E-03	1.46	up
39	SM 8:1;2O/31:0	0.53	-0.91	1.37E-03	1.89	down
40	LPC 22:5-SN1	1.61	0.69	1.40E-03	2.08	up
41	1-(4-chlorophenyl)-2-phenylethan-1-one	0.69	-0.54	1.42E-03	1.73	down
42	PC O-20:4	1.41	0.49	1.51E-03	1.86	up
43	Hydrocortisone	1.39	0.48	1.56E-03	1.53	up
44	3-hydroxy-3-methylpentanedioic acid	2.38	1.25	1.61E-03	1.04	up
45	D-Proline	0.68	-0.56	1.65E-03	1.54	down
46	FRH	1.54	0.63	1.77E-03	1.49	up
47	3-[(4-chlorophenyl)thio]-1-phenylprop-2-en-1-one	0.64	-0.64	1.82E-03	2.19	down
48	Testosterone undecanoate	1.42	0.51	1.83E-03	1.73	up
49	4-Hydroxy-3-methylbenzoic acid	1.40	0.48	1.93E-03	1.81	up
50	Cer 30:0;2O/12:0;O(FA 20:2)	0.25	-2.00	1.94E-03	1.26	down
51	CAR 16:3	1.61	0.69	2.02E-03	1.77	up
52	6-Aminonicotinamide	0.14	-2.88	2.04E-03	1.48	down
53	Oleoyl-L-alpha-lysophosphatidic acid	1.49	0.57	2.11E-03	1.58	up
54	N-Acetyl-L-leucine	0.64	-0.63	2.11E-03	2.19	down
55	(±)13-HpODE	1.38	0.47	2.45E-03	1.54	up
56	SM 12:2;2O/7:0	1.33	0.42	2.68E-03	1.57	up
57	2-Butoxyacetic acid	0.66	-0.61	2.72E-03	1.51	down
58	Cortisol	1.51	0.60	2.86E-03	1.69	up
59	Butylparaben	0.49	-1.03	2.88E-03	1.34	down
60	PC 36:2	0.54	-0.89	2.99E-03	2.15	down
61	β-Cortolone	2.46	1.30	3.11E-03	1.74	up
62	Tangeritin	0.65	-0.61	3.34E-03	1.48	down
63	2-Methoxybenzaldehyde	1.33	0.41	3.34E-03	1.71	up
64	Citrinin	1.32	0.40	3.92E-03	1.48	up
65	Cer 27:0;2O/15:0;O(FA 18:1)	0.37	-1.45	4.06E-03	1.08	down
66	Alanyltyrosine	0.54	-0.88	4.09E-03	1.50	down
67	N1-(4-bromo-2,5-difluorophenyl)-3-methoxybenzamide	0.52	-0.96	4.17E-03	2.02	down
68	Hex3Cer 46:9;2O	0.48	-1.07	4.18E-03	1.37	down
69	Ciprostene	1.52	0.61	4.21E-03	1.60	up
70	Caffeine	0.14	-2.80	4.27E-03	1.38	down
71	JWH 412 N-(5-hydroxypentyl) metabolite	1.39	0.48	4.33E-03	1.48	up
72	DL-Carnitine	0.83	-0.26	4.43E-03	1.84	down
73	Syringic acid	1.96	0.97	4.53E-03	2.09	up
74	PC O-18:3	1.40	0.49	4.58E-03	1.48	up
75	LPC 20:4-SN1	1.29	0.37	4.64E-03	1.71	up
76	Homogentisic Acid	1.93	0.95	4.70E-03	1.47	up
77	AL 8810 Methyl ester	0.67	-0.59	5.05E-03	1.03	down
78	Mag (18:1)	0.52	-0.93	5.14E-03	1.84	down
79	12-Hydroxydodecanoic acid	0.64	-0.65	5.32E-03	1.84	down

80	Progesterone	1.31	0.39	5.44E-03	1.50	up
81	PE 17:1_17:1	0.35	-1.53	5.63E-03	1.36	down
82	PC O-34:9	1.56	0.64	6.03E-03	1.51	up
83	PC O-18:2	1.49	0.58	6.05E-03	1.63	up
84	15-epi Cloprostenol	0.61	-0.70	6.12E-03	1.43	down
85	Benzamidine	1.77	0.82	6.15E-03	1.90	up
86	Dl-Glyceraldehyde3-phosphate	0.75	-0.42	6.55E-03	2.04	down
87	LPC 18:3-SN1	1.35	0.43	6.93E-03	1.36	up
88	Tyrosylalanine	0.53	-0.92	6.94E-03	1.88	down
89	LPC 15:1-SN1	1.31	0.39	7.29E-03	1.62	up
90	PC O-18:1	1.27	0.35	7.37E-03	1.51	up
91	Milbemycin A3 oxime	1.54	0.62	7.47E-03	1.50	up
92	Cer 30:0;2O/12:1;O(FA 18:1)	0.52	-0.95	7.71E-03	1.20	down
93	1,7-bis(3,4-dihydroxyphenyl)heptan-3-one	0.56	-0.83	7.71E-03	1.36	down
94	Nobiletin	0.50	-1.01	7.87E-03	1.38	down
95	LPC 18:0-SN1	1.32	0.40	7.89E-03	1.68	up
96	CAR 26:0	0.53	-0.92	8.11E-03	1.50	down
97	cis-7-Hexadecenoic Acid	1.37	0.45	8.18E-03	1.47	up
98	dUMP	0.64	-0.64	8.44E-03	1.89	down
99	Theophylline	0.14	-2.86	8.53E-03	1.48	down
100	PE O-18:1_20:4	0.50	-0.99	9.07E-03	1.18	down
101	N1-[4-(2-thienylthio)phenyl]-4-chlorobenzamide	221.20	7.79	9.07E-03	2.74	up
102	LPC 16:0	1.44	0.52	9.17E-03	1.71	up
103	Phenylglyoxylic acid	2.29	1.19	9.32E-03	1.69	up
104	Ibuprofen metabolite B	0.03	-5.21	9.32E-03	1.40	down
105	Glu-Gln	1.27	0.34	9.40E-03	1.35	up
106	1,4-dihydroxyheptadec-16-en-2-yl acetate	0.42	-1.27	9.61E-03	1.70	down
107	LPC 22:4	1.63	0.70	9.63E-03	1.74	up
108	2,4-dihydroxyheptadec-16-en-1-yl acetate	1.34	0.42	1.04E-02	1.56	up
109	PE 18:0_18:1	0.61	-0.71	1.06E-02	1.17	down
110	1,3-Dimethyluric acid	0.21	-2.24	1.07E-02	1.38	down
111	N1-(4-cyclohexylphenyl)-2-[(4-methylphenyl)thio]acetamide	1.85	0.89	1.09E-02	1.63	up
112	PE 18:1_20:5	0.38	-1.40	1.11E-02	1.23	down
113	Eicosapentaenoic acid ethyl ester	1.35	0.43	1.13E-02	1.59	up
114	2-Aminobenzenesulfonic acid	2.99	1.58	1.17E-02	1.91	up
115	O-7460	1.36	0.45	1.18E-02	1.30	up
116	PC O-38:10	1.56	0.64	1.18E-02	1.30	up
117	LNH	0.14	-2.88	1.19E-02	1.21	down
118	Estrone	0.76	-0.40	1.19E-02	1.39	down
119	3-methyl-5-oxo-5-(4-toluidino)pentanoic acid	1.29	0.37	1.20E-02	1.41	up
120	L-5-Hydroxytryptophan	1.44	0.53	1.21E-02	1.54	up
121	LysoPE 18:2	1.45	0.54	1.23E-02	1.55	up
122	alpha-Benzylsuccinic acid	1.29	0.37	1.23E-02	1.50	up

123	Lysops 22:5	1.47	0.56	1.25E-02	1.65	up
124	SM 36:2;2O	1.39	0.47	1.27E-02	1.39	up
125	Lysopc 18:1	1.60	0.68	1.27E-02	1.13	up
126	Methionine	1.34	0.42	1.30E-02	1.36	up
127	2-[5-(2-hydroxypropyl)oxolan-2-yl]propanoic acid	1.47	0.56	1.35E-02	1.42	up
128	SM 9:1;2O/38:2	0.52	-0.94	1.36E-02	1.58	down
129	Prostaglandin K2	1.47	0.55	1.36E-02	1.46	up
130	9-Oxo-10(E),12(E)-octadecadienoic acid	1.26	0.33	1.37E-02	1.27	up
131	Mevalonic acid	0.62	-0.69	1.40E-02	1.14	down
132	(±)-Abscisic acid	1.65	0.72	1.41E-02	1.68	up
133	PC 19:2_18:4	0.72	-0.47	1.42E-02	1.12	down
134	PC O-36:9	1.72	0.78	1.42E-02	1.24	up
135	Cinnamyl alcohol	0.06	-4.08	1.42E-02	1.24	down
136	Phloroglucinol	0.76	-0.39	1.44E-02	1.27	down
137	PE 18:1_22:6	0.65	-0.62	1.48E-02	1.43	down
138	Biocytin	1.91	0.94	1.51E-02	1.18	up
139	D-δ-Tocopherol	1.20	0.27	1.60E-02	1.26	up
140	LPC 18:2-SN1	1.32	0.40	1.60E-02	1.52	up
141	Gentisic acid	1.91	0.94	1.63E-02	1.57	up
142	LPC 20:3-SN1	1.30	0.38	1.64E-02	1.46	up
143	3-Methyladipic acid	0.76	-0.40	1.67E-02	1.27	down
144	gamma-Nonanolactone	0.76	-0.40	1.69E-02	1.00	down
145	Tetrahydrocorticosterone	1.69	0.76	1.74E-02	1.25	up
146	15-OxoEDE	1.25	0.32	1.79E-02	1.31	up
147	3-(methylsulfonyl)-2H-chromen-2-one	0.55	-0.87	1.84E-02	1.14	down
148	11-Deoxy prostaglandin F1β	1.25	0.32	1.88E-02	1.33	up
149	Lysope 14:0	0.57	-0.81	1.89E-02	1.85	down
150	Dehydroepiandrosterone	1.64	0.71	1.90E-02	1.53	up
151	Androsterone glucuronide	1.50	0.58	1.97E-02	1.70	up
152	4-hydroxy-3-(3-methylbut-2-en-1-yl)benzoic acid	1.46	0.55	2.00E-02	1.56	up
153	PC O-16:0	1.21	0.28	2.04E-02	1.40	up
154	(5-L-Glutamyl)-L-Amino Acid	1.49	0.58	2.04E-02	1.43	up
155	8,12-iso-iPF2α-VI	0.65	-0.61	2.04E-02	1.17	down
156	2-Hydroxy-3-methylbutanoic acid	0.82	-0.29	2.05E-02	1.58	down
157	L-Glutamate	0.80	-0.32	2.08E-02	1.39	down
158	2-{1-[2-(4-benzhydrylpiperazino)-2-oxoethyl]cyclopentyl}acetic acid	0.29	-1.79	2.09E-02	1.33	down
159	TNK	0.27	-1.87	2.10E-02	1.32	down
160	2-(2,6-dimethoxyphenyl)-5,6-dimethoxy-4H-chromen-4-one	0.71	-0.49	2.13E-02	1.47	down
161	4-Butylresorcinol	0.53	-0.91	2.15E-02	1.51	down
162	LPE 22:5	1.39	0.48	2.16E-02	1.04	up
163	PC O-20:1	1.42	0.51	2.20E-02	1.20	up
164	Di(2-ethylhexyl) phthalate	1.50	0.58	2.25E-02	1.28	up

165	LPC 20:2-SN1	1.35	0.43	2.35E-02	1.43	up
166	Norbuprenorphine	1.44	0.53	2.39E-02	1.18	up
167	LPC O-18:1	1.33	0.41	2.39E-02	1.41	up
168	3-Methyl-2-oxobutanoic acid	0.81	-0.31	2.44E-02	1.79	down
169	13-Hpotre(R)	1.47	0.56	2.50E-02	1.31	up
170	Biotin	1.22	0.28	2.55E-02	1.20	up
171	PC O-40:6	1.23	0.30	2.59E-02	1.58	up
172	PC O-20:2	1.37	0.46	2.60E-02	1.40	up
173	PC 32:2	2.09	1.07	2.63E-02	1.26	up
174	PC 34:7_34:8	1.49	0.57	2.69E-02	1.46	up
175	N-METHYL (-)EPHEDRINE	0.23	-2.12	2.82E-02	1.25	down
176	1,4-dihydroxy-1,4-dimethyl-7-(propan-2-ylidene)-decahydroazulen-6-one	0.64	-0.64	2.86E-02	1.04	down
177	Phellamurin	0.43	-1.20	2.89E-02	1.42	down
178	Ergosterol	1.36	0.45	2.89E-02	1.21	up
179	SM 8:1;2O/24:1	1.78	0.83	2.91E-02	1.12	up
180	mesityl(piperidin-4-yl)methanone hydrochloride	0.33	-1.61	2.97E-02	1.24	down
181	5-Sulfosalicylic acid	0.60	-0.73	3.00E-02	1.24	down
182	SM 8:1;2O/26:7	1.25	0.33	3.04E-02	1.18	up
183	1,7-Dimethyluric acid	0.42	-1.25	3.06E-02	1.14	down
184	N1-[1-(2-furylcarbonyl)-4-piperidyl]benzamide	1.81	0.86	3.07E-02	1.44	up
185	2-(2-carboxy-2-methylpropyl)-4,6-dimethylbenzoic acid	1.66	0.73	3.09E-02	1.51	up
186	3-Acetyl-11-keto-β-boswellic acid	1.24	0.30	3.09E-02	1.15	up
187	4-Methylvaleric Acid	0.65	-0.62	3.10E-02	1.29	down
188	SM 8:1;2O/22:0	1.52	0.60	3.14E-02	1.13	up
189	LPC 18:1-SN1	1.21	0.28	3.15E-02	1.17	up
190	CAR 16:1	1.39	0.48	3.18E-02	1.16	up
191	Milbemycin A4 oxime	1.42	0.51	3.19E-02	1.16	up
192	4-Hexyloxyaniline	0.30	-1.75	3.19E-02	1.25	down
193	Paraxanthine	0.25	-1.98	3.19E-02	1.09	down
194	13,14-dihydro-15-keto Prostaglandin A2	1.27	0.34	3.33E-02	1.20	up
195	RQH	0.43	-1.21	3.33E-02	1.70	down
196	L-Thyroxine	1.28	0.35	3.34E-02	1.38	up
197	PC 18:5_18:5	1.22	0.28	3.38E-02	1.47	up
198	Piperine	0.22	-2.16	3.38E-02	1.47	down
199	Tetrahydroaldosterone	0.43	-1.21	3.40E-02	1.31	down
200	Sodium cholate	1.23	0.30	3.46E-02	1.35	up
201	N1-(3-amino-4-chlorophenyl)-2-[2,4-di(tert-pentyl)phenoxy]acetamide	1.38	0.46	3.48E-02	1.01	up
202	LPC O-16:0	1.33	0.41	3.50E-02	1.37	up
203	Glycodeoxycholic acid	0.48	-1.06	3.51E-02	1.22	down
204	Gamma-Caprolactone	0.72	-0.47	3.51E-02	1.15	down
205	LPC O-16:2	1.30	0.38	3.53E-02	1.07	up
206	Epitestosterone glucuronide	1.37	0.46	3.59E-02	1.39	up

207	1,6-dihydroxy-3-methoxy-8-methyl-9H-xanthen-9-one	0.48	-1.06	3.59E-02	1.36	down
208	CAR 18:3	1.51	0.60	3.65E-02	1.41	up
209	β-Muricholic acid	1.64	0.71	3.68E-02	1.13	up
210	Lysopc 16:2 (2N Isomer)	1.43	0.52	3.77E-02	1.41	up
211	Methyl EudesMate	0.40	-1.31	3.79E-02	1.49	down
212	2-Methoxyestradiol (2-MeOE2)	1.26	0.33	3.82E-02	1.17	up
213	Artemisinin	1.31	0.39	3.82E-02	1.47	up
214	LPC 20:1	1.46	0.54	3.83E-02	1.23	up
215	methyl 4-methyl-2-oxo-2H-pyran-6-carboxylate	2.76	1.47	3.87E-02	1.63	up
216	QKK	0.60	-0.74	3.89E-02	1.23	down
217	(12Z)-9,10,11-trihydroxyoctadec-12-enoic acid	1.56	0.64	3.96E-02	1.07	up
218	L-Glutamic acid	0.76	-0.39	4.07E-02	1.23	down
219	Tetracycline	0.83	-0.27	4.10E-02	1.04	down
220	R-1 Methanandamide phosphate	0.65	-0.62	4.18E-02	1.30	down
221	4-Hydroxybenzophenone	1.44	0.52	4.21E-02	1.16	up
222	3-methoxy-2-phenyl-4H-furo[2,3-h]chromen-4-one	0.20	-2.30	4.29E-02	1.02	down
223	Monoolein	0.40	-1.33	4.32E-02	1.41	down
224	L-Cysteine-glutathione gisulfide	1.71	0.77	4.32E-02	1.31	up
225	N-Acetyl-aspartic acid	1.40	0.49	4.33E-02	1.19	up
226	(1R,2R)-trans-N-Boc-1,2-cyclohexanediamine	0.55	-0.87	4.38E-02	1.75	down
227	LysoPC 20:2	1.69	0.76	4.41E-02	1.17	up
228	PC 15:0_18:2	0.62	-0.69	4.41E-02	1.26	down
229	HPK	0.47	-1.08	4.43E-02	1.14	down
230	Thromoboxane B1	0.44	-1.18	4.47E-02	1.18	down
231	Vitamin A	1.38	0.46	4.49E-02	1.14	up
232	CAR 24:0	0.58	-0.78	4.50E-02	1.42	down
233	NAD^+	1.32	0.40	4.50E-02	1.15	up
234	Bicyclo Prostaglandin E2	1.34	0.43	4.52E-02	1.32	up
235	JWH 250 N-pentanoic acid metabolite	0.31	-1.67	4.54E-02	1.02	down
236	Prostaglandin F2α-1-glyceryl ester	0.26	-1.93	4.56E-02	1.17	down
237	O-Desmethylnaproxen	0.55	-0.87	4.63E-02	1.34	down
238	RNK	0.31	-1.69	4.65E-02	1.19	down
239	10-Hydroxydecanoic acid	0.66	-0.60	4.91E-02	1.42	down
240	Cafestol	1.43	0.51	4.96E-02	1.05	up
241	Lysopc 18:2	1.37	0.45	4.96E-02	1.14	up
242	Gamma-Glu-Leu	0.74	-0.43	4.97E-02	1.09	down
243	SM 9:1;2O/30:8	1.37	0.45	4.99E-02	1.10	up
