# Supplementary material for: Multi-Omics Mechanism of Chronic Gout Arthritis and Discovery of the Thyroid Hormone–AMPK–Taurine Metabolic Axis
Source: Cells. 2025 Dec 25;15(1):41. doi: 10.3390/cells15010041 (PMC12785424; doi:10.3390/cells15010041)
Supplement: Supplementary file 1 [file cells-15-00041-s001.zip › Differential metabolites in CGA vs control comparison.pdf]

Differential metabolites in CGA vs control comparison

No.	Metabolite	FC	log2FC	P	VIP	Up.Down
1	(2R,3S,4S,5R,6R)-2-(hydroxymethyl)-6-(propan-2-yloxy)oxane-3,4,5-triol	3.14	1.65	3.22E-16	2.09	up
2	4-oxododecanedioic acid	0.09	-3.46	8.41E-16	2.39	down
3	Monobutyl phthalate	3.87	1.95	1.09E-15	2.24	up
4	Milbemectin A3	5.01	2.33	1.02E-14	2.11	up
5	AL 8810 Methyl ester	0.24	-2.05	5.90E-14	2.52	down
6	Ecdysterone	0.29	-1.78	1.74E-13	1.97	down
7	6beta-Naltrexol-d3	4.14	2.05	3.50E-13	2.15	up
8	LNH	0.03	-4.99	4.91E-13	2.40	down
9	5α-Dihydrotestosterone glucuronide	0.33	-1.60	8.24E-13	2.31	down
10	3-hydroxy-3-methylpentanedioic acid	20.06	4.33	8.46E-13	2.32	up
11	2-Methoxyresorcinol	0.18	-2.51	1.24E-12	2.16	down
12	17alpha-Ethinyl estradiol	0.21	-2.23	1.31E-12	2.32	down
13	HPK	0.19	-2.40	2.85E-12	2.14	down
14	(2R)-2-[(2R,5S)-5-[(2S)-2-hydroxybutyl]oxolan-2-yl]propanoic acid	0.14	-2.80	8.54E-12	2.17	down
15	Thromoboxane B1	0.14	-2.79	3.66E-11	2.26	down
16	2-(tert-butyl)-1,3-thiazolane-4-carboxylic acid	0.12	-3.06	3.85E-11	1.96	down
17	6-(3-hydroxybutan-2-yl)-5-(hydroxymethyl)-4-methoxy-2H-pyran-2-one	0.42	-1.27	4.57E-11	2.06	down
18	Asaraldehyde	0.36	-1.46	9.31E-11	2.25	down
19	PC O-36:7	0.38	-1.41	1.24E-10	2.00	down
20	PC 19:2_18:5	8.42	3.07	1.54E-10	2.32	up
21	Butylparaben	0.31	-1.67	2.85E-10	2.10	down
22	JWH 250 N-pentanoic acid metabolite	0.10	-3.34	3.53E-10	2.19	down
23	Prostaglandin F2α-1-glyceryl ester	0.07	-3.92	5.18E-10	2.27	down
24	RMK	0.33	-1.58	5.89E-10	2.23	down
25	Sedanolide	0.22	-2.20	1.34E-09	1.81	down
26	N'1-[4-(tert-butyl)benzoyl]-4-(tert-butyl)benzene-1-carbohydrazide	2.62	1.39	1.97E-09	1.88	up
27	Hex3Cer 46:9;2O	0.21	-2.22	2.26E-09	2.31	down
28	Norbuprenorphine	22.54	4.49	2.33E-09	2.20	up
29	Delta-Tridecalactone	0.21	-2.23	2.67E-09	1.96	down
30	4-Hydroxybenzophenone	195.07	7.61	3.54E-09	2.25	up
31	4-methoxy-6-[2-(4-methoxyphenyl)ethyl]-2H-pyran-2-one	7.11	2.83	4.04E-09	2.23	up
32	N'2-(2-hydroxybenzylidene)-5-nitrofuran-2-carbohydrazide	8.89	3.15	4.37E-09	2.19	up
33	3-(3-morpholinopropyl)-2-(2-pyridinyl)-2,3-dihydro-4(1H)-quinazolinone	0.51	-0.98	4.86E-09	2.11	down
34	NAD^+	12.49	3.64	5.23E-09	2.30	up

35	Lysopc 18:1	16.11	4.01	5.45E-09	2.46	up
36	mesityl(piperidin-4-yl)methanone hydrochloride	0.10	-3.34	5.62E-09	2.31	down
37	RNK	0.07	-3.83	6.01E-09	2.32	down
38	Kahweol	0.25	-1.97	6.21E-09	1.80	down
39	p-Mentha-1,3,8-triene	0.12	-3.07	6.77E-09	2.25	down
40	N-METHYL (-)EPHEDRINE	0.07	-3.93	7.02E-09	2.28	down
41	4-(pentyloxy)benzene-1-carbohydrazide	0.17	-2.57	7.21E-09	2.20	down
42	3-phenyl-5-[3-(trifluoromethyl)-1H-pyrazol-1-yl]-1,2,4-thiadiazole	5.03	2.33	7.27E-09	2.19	up
43	Spinosyn A	11.48	3.52	7.91E-09	2.36	up
44	1,3-bis(4-methoxybenzyl)-2-phenylhexahydropyrimidine	0.54	-0.89	7.93E-09	1.77	down
45	Ginsenoside Rg3	0.09	-3.55	8.74E-09	2.23	down
46	2-Methylbutyl beta-D-glucopyranoside	2.39	1.26	8.88E-09	1.65	up
47	PC O-36:8	13.54	3.76	9.85E-09	2.44	up
48	4-Hexyloxyaniline	0.09	-3.48	1.03E-08	2.27	down
49	SM 8:1;2O/26:7	3.22	1.69	1.15E-08	2.20	up
50	3-methoxy-2-phenyl-4H-furo[2,3-h]chromen-4-one	0.56	-0.82	1.16E-08	1.23	down
51	Indole-3-lactic acid	5.46	2.45	1.28E-08	2.51	up
52	SM 9:1;2O/30:8	9.94	3.31	1.30E-08	2.37	up
53	D-Ribose	2.11	1.08	1.31E-08	2.13	up
54	QKK	0.20	-2.33	1.39E-08	2.48	down
55	Benzoic acid	8.75	3.13	1.46E-08	2.25	up
56	Lysopc 18:3	6.69	2.74	1.49E-08	2.22	up
57	Milbemycin A4 oxime	10.19	3.35	1.50E-08	2.37	up
58	6-(7-methyloctyl)-1H,3H,4H,6H-furo[3,4-c]furan-1-one	0.10	-3.28	1.51E-08	2.17	down
59	Tangeritin	0.56	-0.84	1.56E-08	1.94	down
60	5-acetyl-2,6-dimethyl-1,2,3,4-tetrahydropyridin-4-one	0.11	-3.14	1.68E-08	2.10	down
61	3-(2-methylpropyl)-octahydropyrrolo[1,2-a]pyrazine-1,4-dione	0.16	-2.69	1.92E-08	2.10	down
62	Di(2-ethylhexyl) phthalate	10.84	3.44	2.09E-08	2.08	up
63	Testosterone glucuronide	4.32	2.11	2.41E-08	2.09	up
64	Fasciculic acid C	10.02	3.33	2.57E-08	2.39	up
65	Tetrahydroaldosterone	0.17	-2.52	2.63E-08	2.16	down
66	gamma-Nonanolactone	0.50	-1.01	2.70E-08	1.85	down
67	SM 9:1;2O/24:6	11.12	3.47	2.75E-08	2.15	up
68	morphine-d3	14.25	3.83	2.85E-08	2.07	up
69	(11E,15Z)-9,10,13-trihydroxyoctadeca-11,15-dienoic acid	0.43	-1.23	3.03E-08	1.78	down
70	Bicyclo Prostaglandin E2	24.10	4.59	3.06E-08	2.19	up
71	2-Methylpentanedioic acid	9.05	3.18	3.07E-08	2.19	up
72	LPC 32:9-SN2	4.14	2.05	3.63E-08	2.49	up
73	Taurocholic acid	0.12	-3.05	3.81E-08	1.75	down

74	8-Bromoguanosine	20.19	4.34	4.33E-08	2.14	up
75	SM 8:1;2O/13:1	4.44	2.15	4.50E-08	1.92	up
76	3-Phenyllactic acid	2.53	1.34	4.85E-08	2.17	up
77	L-cysteine	6.69	2.74	4.90E-08	2.02	up
78	Thromboxane B2-biotin	6.56	2.71	5.31E-08	2.02	up
79	PC O-38:10	6.61	2.73	5.81E-08	2.39	up
80	LysoPC 20:2	30.42	4.93	6.14E-08	2.41	up
81	3-[(4-hydroxyphenyl)methyl]-octahydropyrrolo[1,2-a]pyrazine-1,4-dione	5.37	2.42	6.62E-08	2.07	up
82	TNK	0.10	-3.34	6.68E-08	2.27	down
83	SM 8:1;2O/28:7	2.19	1.13	1.10E-07	2.22	up
84	PC O-36:9	14.42	3.85	1.15E-07	2.37	up
85	Lithocholic Acid	0.19	-2.42	1.25E-07	1.78	down
86	methyl 3,4,5-trihydroxycyclohex-1-ene-1-carboxylate	0.16	-2.60	1.32E-07	2.21	down
87	2-(2-oxo-2-{[2-(2-oxo-1-imidazolidinyl)ethyl]amino}ethoxy)acetic acid	3.00	1.58	1.75E-07	1.99	up
88	2-[5-(2-hydroxypropyl)oxolan-2-yl]propanoic acid	5.08	2.34	1.95E-07	2.15	up
89	4-morpholinobenzoic acid	0.22	-2.20	2.60E-07	2.05	down
90	4-tert-Amylphenol	0.30	-1.72	3.14E-07	2.14	down
91	N-benzyl-N-isopropyl-N'-(4-isopropylphenyl)thiourea	3.11	1.64	3.14E-07	2.21	up
92	3-Indoleacrylic acid	2.51	1.33	3.26E-07	1.72	up
93	O-7460	4.52	2.18	3.73E-07	1.75	up
94	N-Acetylcytidine	1.84	0.88	4.64E-07	1.96	up
95	5-(2,5-dihydroxyhexyl)oxolan-2-one	1.29	0.37	4.68E-07	1.78	up
96	PC 17:2_17:2	7.53	2.91	5.04E-07	2.13	up
97	4-{3-[(3,4-dihydroxyphenyl)methyl]-2-methylbutyl}benzene-1,2-diol	0.34	-1.54	5.05E-07	2.00	down
98	Feruloyl Putrescine	2.07	1.05	6.11E-07	1.91	up
99	15-epi Cloprostenol	0.54	-0.90	6.16E-07	1.80	down
100	NSI-189	4.28	2.10	7.25E-07	2.24	up
101	Phenylacetaldehyde	2.27	1.18	7.31E-07	2.03	up
102	1-Methylguanine	1.38	0.46	7.37E-07	2.02	up
103	Tolterodine	0.31	-1.70	9.41E-07	1.95	down
104	Spiculisporic Acid	0.50	-1.01	1.21E-06	1.43	down
105	2-Phenylpropionic acid	1.71	0.78	1.38E-06	1.50	up
106	ST 24:2;O4	0.28	-1.86	1.43E-06	1.81	down
107	3-(4-hydroxy-3-methoxyphenyl)propanoic acid	3.64	1.86	1.44E-06	1.95	up
108	3-Hydroxybenzoic acid	2.26	1.17	1.45E-06	1.58	up
109	Cyclohexaneacetic acid	2.68	1.42	1.51E-06	2.13	up
110	PC O-34:8	4.13	2.05	1.51E-06	1.98	up
111	R-1 Methanandamide phosphate	0.39	-1.37	1.59E-06	2.06	down
112	3,5-Dihydroxybenzoic acid	3.36	1.75	1.68E-06	1.91	up
113	SM 9:1;2O/26:7	1.90	0.92	2.26E-06	1.96	up
114	Perfluorooctanoic acid (PFOA)	0.02	-5.39	2.52E-06	1.55	down

115	6-Hydroxymelatonin	0.27	-1.88	3.25E-06	2.13	down
116	Glycodeoxycholic acid	0.21	-2.28	3.47E-06	2.01	down
117	Levothyroxine	1.75	0.81	4.40E-06	1.34	up
118	1,7-bis(3,4-dihydroxyphenyl)heptan-3-one	0.70	-0.52	4.48E-06	1.11	down
119	Valproic acid	2.90	1.53	5.37E-06	1.85	up
120	Tacrolimus	1.77	0.82	5.69E-06	1.79	up
121	Prolylleucine	1.41	0.50	6.10E-06	1.34	up
122	Ginsenoside Rg2	1.50	0.58	6.38E-06	1.67	up
123	(12Z)-9,10,11-trihydroxyoctadec-12-enoic acid	3.66	1.87	6.43E-06	2.05	up
124	D-Xylonic Acid	2.37	1.24	7.44E-06	2.14	up
125	Spermidine	2.52	1.33	8.29E-06	1.70	up
126	Piperine	0.14	-2.79	8.33E-06	1.69	down
127	4-Methoxycinnamic Acid	0.46	-1.12	8.44E-06	1.43	down
128	Cannabichromevarin	0.47	-1.10	8.62E-06	1.57	down
129	Azelaic acid	1.94	0.95	9.14E-06	1.35	up
130	β-Cortolone	4.67	2.22	9.77E-06	2.05	up
131	2-(cyclopropylcarbonyl)-3-(4-fluoroanilino)acrylonitrile	0.56	-0.83	1.01E-05	1.36	down
132	RQH	0.47	-1.10	1.11E-05	1.53	down
133	4-methylphenyl 1-ethyl-3-methyl-1H-pyrazole-5-carbothioate	3.48	1.80	1.12E-05	1.94	up
134	SM 8:1;2O/13:0	1.26	0.33	1.14E-05	1.21	up
135	DL-α-Aminocaprylic acid	15.21	3.93	1.25E-05	1.84	up
136	3-oxoindane-1-carboxylic acid	1.71	0.77	1.34E-05	1.37	up
137	NKK	0.66	-0.59	1.41E-05	1.53	down
138	3-Methylindole	0.38	-1.38	1.47E-05	1.86	down
139	Oxoadipic Acid	2.22	1.15	1.64E-05	1.89	up
140	5,6-dimethyl-4-oxo-4H-pyran-2-carboxylic acid	7.92	2.99	1.79E-05	1.85	up
141	4-Methylvaleric Acid	0.45	-1.17	1.79E-05	1.71	down
142	N6-Succinyl Adenosine	1.49	0.58	1.91E-05	1.79	up
143	4-Hydroxybenzoic acid	5.28	2.40	2.04E-05	1.91	up
144	Dl-3,4-Dihydroxymandelic Acid	5.28	2.40	2.04E-05	1.91	up
145	PC 19:0_20:4	1.26	0.34	2.10E-05	1.21	up
146	2-methyl-1,2-dihydrophthalazin-1-one	1.56	0.64	2.41E-05	1.23	up
147	SM 8:1;2O/9:0	2.10	1.07	2.83E-05	1.38	up
148	1-(3-methoxy-2-nitrostyryl)pyrrolidine	0.46	-1.13	2.96E-05	1.45	down
149	ENK	1.66	0.73	3.10E-05	1.23	up
150	Dextromethorphan hydrobromide	0.45	-1.16	3.46E-05	1.93	down
151	Dimetghyl 4-Hydroxyisophthalate	1.74	0.80	3.61E-05	1.57	up
152	MGMG (18:2)	0.50	-1.00	3.79E-05	1.78	down
153	THC	1.25	0.32	3.86E-05	1.25	up
154	Octadecanamine	0.54	-0.89	3.92E-05	1.39	down
155	N-Formylkynurenine	0.56	-0.83	4.10E-05	1.23	down
156	(±)13-HpODE	1.64	0.72	4.38E-05	1.50	up

157	TLK	1.32	0.40	4.51E-05	1.58	up
158	2-Methoxyestradiol (2-MeOE2)	1.76	0.81	4.58E-05	1.57	up
159	Citrinin	1.51	0.60	4.61E-05	1.47	up
160	Phenylglyoxylic acid	4.83	2.27	5.43E-05	2.11	up
161	SM 9:1;2O/32:9	4.15	2.05	5.61E-05	1.87	up
162	cis,cis-Muconic acid	2.03	1.02	5.63E-05	1.66	up
163	Phloroglucinol	0.61	-0.70	5.92E-05	1.58	down
164	5α-Tetrahydrocortisol	3.76	1.91	6.00E-05	1.93	up
165	Prostaglandin G2	1.59	0.67	7.51E-05	1.71	up
166	4-Hydroxyisoleucine	1.29	0.36	7.85E-05	1.42	up
167	5,6-dimethoxy-2-(2-methoxyphenyl)-4H-chromen-4-one	1.28	0.36	8.41E-05	1.36	up
168	PC O-34:9	2.16	1.11	9.43E-05	1.42	up
169	N-Acetyl-DL-tryptophan	1.65	0.73	9.51E-05	1.29	up
170	Homovanillic acid	1.77	0.82	9.71E-05	1.79	up
171	O-Desmethylnaproxen	0.60	-0.75	1.01E-04	1.42	down
172	N2,N2-Dimethylguanosine	1.42	0.50	1.09E-04	1.96	up
173	(1R,2R)-trans-N-Boc-1,2-cyclohexanediamine	0.56	-0.83	1.19E-04	1.64	down
174	Lysope 14:0	0.57	-0.80	1.21E-04	1.56	down
175	Diphenylamine	2.84	1.50	1.21E-04	1.74	up
176	Gentisic acid	3.32	1.73	1.36E-04	1.86	up
177	Syringic acid	3.44	1.78	1.38E-04	2.16	up
178	3-hydroxy-1,5-diphenylpentan-1-one	0.43	-1.23	1.61E-04	1.51	down
179	CAR 8:2	5.61	2.49	1.80E-04	1.95	up
180	Homogentisic Acid	3.13	1.65	1.88E-04	1.37	up
181	L-beta-Imidazolelactic acid	1.68	0.75	1.91E-04	1.46	up
182	Tyramine	2.54	1.35	2.22E-04	1.42	up
183	Lysopg 18:1	0.49	-1.02	2.28E-04	1.25	down
184	Acetyl-L-carnitine	1.60	0.68	2.67E-04	1.35	up
185	Glycoursodeoxycholic acid	0.43	-1.23	2.80E-04	1.30	down
186	Normorphine	0.39	-1.37	2.97E-04	1.16	down
187	8-Aminooctanoic acid	1.61	0.68	3.15E-04	1.40	up
188	Andrographolide	0.27	-1.89	3.19E-04	1.22	down
189	LPC O-15:0	0.54	-0.89	3.24E-04	1.42	down
190	Pseudouridine	1.82	0.86	3.27E-04	1.25	up
191	PC 8:0_9:0	2.18	1.12	3.44E-04	1.41	up
192	Mag (18:1)	0.57	-0.81	3.59E-04	1.35	down
193	4-hydroxy-3-(3-methylbut-2-en-1-yl)benzoic acid	2.34	1.23	3.85E-04	1.45	up
194	Dehydroepiandrosterone	2.35	1.23	3.89E-04	1.73	up
195	5-Hydroxyindole-3-acetic acid	1.36	0.45	4.32E-04	1.39	up
196	3-Oxo-7alpha,12alpha-hydroxy-5beta-cholanoic acid	0.65	-0.63	4.79E-04	1.44	down
197	Prunin	0.56	-0.85	4.83E-04	1.11	down
198	Benzamidine	2.15	1.11	4.97E-04	1.83	up
199	3-(3-Methoxyphenyl)propionic acid	1.66	0.73	5.71E-04	1.46	up

200	MAG (18:2)	0.70	-0.52	6.02E-04	1.34	down
201	CAR 7:0	3.46	1.79	6.20E-04	1.39	up
202	2-[(3S)-1-Benzyl-3-pyrrolidinyl]-1,3-benzothiazole	0.48	-1.07	6.31E-04	1.56	down
203	3-(methylsulfonyl)-2H-chromen-2-one	0.53	-0.93	6.63E-04	1.15	down
204	N1-(4-cyclohexylphenyl)-2-[(4-methylphenyl)thio]acetamide	2.61	1.38	6.85E-04	1.59	up
205	PC O-38:9	9.48	3.25	7.43E-04	2.00	up
206	2-Arachidonyl Glycerol ether	0.71	-0.50	7.93E-04	1.30	down
207	L-(-)-Glyceric acid	1.23	0.30	8.09E-04	1.05	up
208	Hesperetin	0.63	-0.66	8.14E-04	1.80	down
209	Phellamurin	0.55	-0.85	8.32E-04	1.26	down
210	7-[(3,3-dimethyloxiran-2-yl)methoxy]-6-methoxy-2H-chromen-2-one	4.26	2.09	8.37E-04	1.39	up
211	Royal jelly acid	2.05	1.04	8.62E-04	1.39	up
212	Suberic acid	1.40	0.48	8.82E-04	1.15	up
213	Cotinine	29.72	4.89	9.33E-04	1.46	up
214	Dihydrothymine	1.80	0.85	9.48E-04	1.15	up
215	N3,N4-Dimethyl-L-arginine	1.31	0.38	9.88E-04	1.38	up
216	PC 36:5	0.61	-0.72	1.04E-03	1.42	down
217	2-Arachidonoyl glycerol	0.50	-1.00	1.06E-03	1.34	down
218	N-acetyl-L-ornithine	2.49	1.32	1.23E-03	1.68	up
219	Undecanedioic acid	1.52	0.61	1.23E-03	1.36	up
220	Taurochenodeoxycholic acid	0.36	-1.49	1.25E-03	1.49	down
221	Vitamin A	1.74	0.80	1.29E-03	1.41	up
222	LPC O-14:0	0.52	-0.94	1.29E-03	1.13	down
223	11-Deoxy prostaglandin F2β	0.71	-0.49	1.36E-03	1.46	down
224	Nonanoic acid	1.81	0.86	1.41E-03	1.40	up
225	N1-[1-(2-furylcarbonyl)-4-piperidyl]benzamide	3.64	1.86	1.54E-03	1.48	up
226	2-(2-carboxy-2-methylpropyl)-4,6-dimethylbenzoic acid	2.39	1.26	1.60E-03	1.62	up
227	(±)-Abscisic acid	2.05	1.03	1.64E-03	1.56	up
228	N4-Acetylcytidine	1.55	0.63	1.73E-03	1.54	up
229	LPC 22:3-SN1	0.57	-0.82	1.74E-03	1.07	down
230	gamma-Glutamyltyrosine	1.21	0.28	1.79E-03	1.19	up
231	D-Gluconic acid	1.43	0.52	1.81E-03	1.34	up
232	5-[(10Z)-14-(3,5-dihydroxyphenyl)tetradec-10-en-1-yl]benzene-1,3-diol	17.65	4.14	1.84E-03	1.37	up
233	Adenosine	29.30	4.87	2.07E-03	1.80	up
234	Emamectin benzoate	0.27	-1.89	2.09E-03	1.23	down
235	Hexanoylcarnitine	2.66	1.41	2.20E-03	1.42	up
236	6-Keto-prostaglandin f1alpha	0.58	-0.79	2.44E-03	1.08	down
237	L-Carnitine	1.58	0.66	2.51E-03	1.36	up
238	Ethyl paraben	2.08	1.06	2.52E-03	1.40	up
239	4'-methyl Acetyl fentanyl-d5	1.80	0.85	2.54E-03	1.19	up

240	Docosanoic acid	0.61	-0.72	2.71E-03	1.07	down
241	N-(6-methoxypyridin-3-yl)thiophene-2-carboxamide	1.26	0.33	2.87E-03	1.23	up
242	Serotonin	2.61	1.38	2.88E-03	1.31	up
243	Glutaconic acid	1.74	0.80	2.93E-03	1.31	up
244	2,3-dihydroxypropyl 12-methyltridecanoate	1.25	0.32	3.29E-03	1.05	up
245	3-amino-1H-pyrazolo[4,3-c]pyridine-4,6-diol	1.27	0.35	3.39E-03	1.19	up
246	Decanoic acid	1.98	0.99	3.44E-03	1.30	up
247	Corey Lactone Diol	1.96	0.97	3.46E-03	1.48	up
248	Kynurenic acid O-hexside	2.47	1.30	3.60E-03	1.34	up
249	Fenpropimorph	7.57	2.92	3.78E-03	1.22	up
250	WKK	2.30	1.20	3.87E-03	1.32	up
251	2-(2,6-dimethoxyphenyl)-5,6-dimethoxy-4H-chromen-4-one	0.77	-0.37	3.87E-03	1.12	down
252	Corticosterone	0.74	-0.44	4.19E-03	1.40	down
253	LPC O-16:2	1.46	0.55	4.25E-03	1.07	up
254	Methionine	1.41	0.49	4.26E-03	1.12	up
255	4-(allyloxy)-1,2-dihydroquinolin-2-one	0.66	-0.59	4.34E-03	1.38	down
256	3-morpholino-5,6-diphenylpyridazine-4-carbonitrile	0.51	-0.96	4.34E-03	1.21	down
257	ST 24:1;O4;S	0.36	-1.49	4.37E-03	1.07	down
258	(2S)-2-(2-hydroxypropan-2-yl)-2H,3H,7H-furo[3,2-g]chromen-7-one	0.79	-0.33	4.63E-03	1.33	down
259	6-Pentyl-2H-pyran-2-one	0.82	-0.29	4.89E-03	1.35	down
260	Gemifloxacin	0.70	-0.52	5.18E-03	1.30	down
261	N-Acetylalanine	1.64	0.71	5.23E-03	1.61	up
262	Biotin	1.30	0.38	5.24E-03	1.06	up
263	Glycochenodeoxycholic acid	0.53	-0.91	5.25E-03	1.21	down
264	(2E,4E)-N-(2-methylpropyl)dodeca-2,4-dienamide	6.10	2.61	5.33E-03	1.23	up
265	11(Z)-Eicosenoic acid	0.60	-0.73	5.40E-03	1.02	down
266	2,5-Dimethylphenol	0.19	-2.40	5.61E-03	1.03	down
267	L-Ascorbic acid 2-sulfate	1.55	0.64	5.81E-03	1.15	up
268	2,6-Dihydroxypurine	5.27	2.40	6.41E-03	1.45	up
269	LPC 22:5-SN1	1.51	0.59	6.62E-03	1.15	up
270	1-benzyl-3-(2-methylphenyl)-3,7-dihydro-1H-purine-2,6-dione	0.46	-1.11	6.88E-03	1.30	down
271	L-Thyroxine	1.39	0.47	7.26E-03	1.21	up
272	IDP	1.67	0.74	7.44E-03	1.82	up
273	PC O-22:4	1.71	0.77	7.49E-03	1.33	up
274	2,3,4-Trihydroxybenzoic acid	1.88	0.91	7.79E-03	1.36	up
275	2-Furoic acid	1.44	0.53	7.92E-03	1.23	up
276	12-Hydroxydodecanoic acid	0.70	-0.52	7.99E-03	1.11	down
277	CAR 16:3	1.50	0.59	8.65E-03	1.01	up
278	Hippuric acid	0.58	-0.78	9.35E-03	1.12	down
279	Choline Glycerophosphate	1.24	0.31	9.50E-03	1.22	up
280	Narasin	2.20	1.14	9.62E-03	1.25	up

281	CAR 9:1	7.35	2.88	9.80E-03	1.38	up
282	N1-[4-(2-thienylthio)phenyl]-4-chlorobenzamide	198.53	7.63	1.04E-02	1.86	up
283	trans-Aconitic acid	1.81	0.85	1.13E-02	1.47	up
284	3-(3,4,5-trimethoxyphenyl)propanoic acid	2.64	1.40	1.15E-02	1.55	up
285	LPE O-17:1	0.65	-0.61	1.17E-02	1.32	down
286	Citric acid	1.50	0.58	1.17E-02	1.23	up
287	3-(4-methoxyphenoxy)propanoic acid	8.81	3.14	1.23E-02	1.62	up
288	3-(propan-2-yl)-octahydropyrrolo[1,2-a]pyrazine-1,4-dione	0.69	-0.53	1.26E-02	1.41	down
289	5-chloro-6-(trifluoromethyl)-1,3-dihydro-2H-benzimidazole-2-thione	1.82	0.86	1.34E-02	1.20	up
290	23-Norcholic acid	0.66	-0.60	1.36E-02	1.14	down
291	aminoimidazole carboxamide ribonucleotide	0.76	-0.40	1.38E-02	1.27	down
292	3-(4-chlorophenyl)-5-(methylthio)-1H-pyrazole-4-carbonitrile	2.71	1.44	1.39E-02	2.00	up
293	N-Tetradecanamide	4.46	2.16	1.42E-02	1.11	up
294	Hexadecanamide	7.59	2.92	1.51E-02	1.10	up
295	Taurochenodeoxycholic Acid (sodium salt)	0.59	-0.76	1.54E-02	1.14	down
296	PC O-40:11	1.65	0.73	1.54E-02	1.36	up
297	Kynurenic acid	1.85	0.89	1.64E-02	1.26	up
298	L-Hydroxyproline	2.56	1.36	1.65E-02	1.61	up
299	Stearamide	7.47	2.90	1.76E-02	1.10	up
300	Cystine	1.34	0.43	1.80E-02	1.22	up
301	Xanthosine	7.84	2.97	1.82E-02	1.36	up
302	Methyl indole-3-acetate	4.73	2.24	1.83E-02	1.02	up
303	1-(4-methyl-2-morpholino-1,3-thiazol-5-yl)ethan-1-one	2.95	1.56	1.86E-02	1.14	up
304	1-Methylguanosine	1.43	0.52	1.86E-02	1.25	up
305	Betaine	1.34	0.42	1.90E-02	1.23	up
306	Gly-Phe	0.63	-0.67	1.98E-02	1.50	down
307	cis-Aconitic acid	1.76	0.82	2.00E-02	1.44	up
308	Oleoyl ethylamide	5.89	2.56	2.01E-02	1.03	up
309	Prostaglandin H1	0.78	-0.36	2.03E-02	1.21	down
310	2,3-dinor Prostaglandin E1	0.72	-0.47	2.16E-02	1.20	down
311	N-Acetyl-D-tryptophan	0.76	-0.40	2.17E-02	1.22	down
312	Sphingosine (d18:1)	0.61	-0.71	2.22E-02	1.23	down
313	Gly-Val	0.69	-0.53	2.40E-02	1.08	down
314	LPE O-18:2	0.74	-0.44	2.49E-02	1.22	down
315	Porphobilinogen	1.36	0.45	2.56E-02	1.14	up
316	2-Aminobenzenesulfonic acid	2.01	1.01	2.58E-02	1.25	up
317	17(S)-HpDHA	0.80	-0.32	2.71E-02	1.04	down
318	1,7-bis(4-hydroxyphenyl)heptan-3-one	0.83	-0.26	2.84E-02	1.13	down
319	D-Sphingosine	0.67	-0.59	2.89E-02	1.06	down
320	5-Hydroxyindole	4.77	2.25	3.47E-02	1.52	up
321	L-Cystine	1.29	0.36	3.50E-02	1.00	up

322	gamma-Glutamylleucine	1.24	0.32	3.53E-02	1.10	up
323	Uric acid	1.39	0.48	3.89E-02	1.17	up
324	LPE O-18:1	0.76	-0.40	4.02E-02	1.09	down
325	LPE 22:1	1.98	0.98	4.38E-02	1.39	up
