# Supplementary material for: Multi-Omics Mechanism of Chronic Gout Arthritis and Discovery of the Thyroid Hormone–AMPK–Taurine Metabolic Axis
Source: Cells. 2025 Dec 25;15(1):41. doi: 10.3390/cells15010041 (PMC12785424; doi:10.3390/cells15010041)
Supplement: Supplementary file 1 [file cells-15-00041-s001.zip › Differentially expressed proteins in CGA vs AGA comparison.pdf]

Differentially expressed proteins in CGA vs AGA comparison

No.	Protein	P	FC	log2FC	Up.Down
1	HSPA8	1.32E-07	1.59	0.67	up
2	KDM5D	5.80E-07	2.09	1.07	up
3	GUCY1A2	2.37E-05	0.46	-1.13	down
4	TMOD2	2.98E-05	1.84	0.88	up
5	RACK1	4.18E-05	2.44	1.29	up
6	COL6A3	4.67E-05	1.35	0.43	up
7	ZNF469	5.16E-05	1.81	0.85	up
8	EEF2	5.77E-05	1.79	0.84	up
9	VEGFC	8.24E-05	1.57	0.65	up
10	KDM4C	8.68E-05	2.29	1.20	up
11	CD44	9.49E-05	1.26	0.33	up
12	CDK5RAP2	1.02E-04	0.67	-0.58	down
13	KHSRP	1.38E-04	0.62	-0.68	down
14	OPTN	4.43E-04	3.04	1.60	up
15	TSKU	6.85E-04	4.30	2.10	up
16	PPA2	7.21E-04	0.41	-1.27	down
17	NT5DC2	9.59E-04	1.64	0.72	up
18	KIF13B	1.04E-03	0.80	-0.31	down
19	FAP	1.09E-03	1.35	0.44	up
20	ALB	1.09E-03	0.42	-1.26	down
21	ZBTB20	1.55E-03	1.41	0.50	up
22	CLSTN1	1.59E-03	1.35	0.43	up
23	B2M	1.60E-03	1.25	0.32	up
24	AK1	1.81E-03	2.36	1.24	up
25	SELL	2.07E-03	1.21	0.28	up
26	LDHC	2.15E-03	1.29	0.37	up
27	ADGRL4	2.28E-03	1.59	0.67	up
28	GGT1	2.82E-03	1.80	0.85	up
29	SERPINA5	2.96E-03	1.63	0.70	up
30	RPL3	3.27E-03	2.27	1.18	up
31	SPOCK1	3.87E-03	1.29	0.37	up
32	SEMA4A	4.13E-03	0.76	-0.40	down
33	GLO1	4.18E-03	0.44	-1.20	down
34	RAB5A	4.59E-03	2.74	1.45	up
35	MAN1A1	5.21E-03	0.80	-0.32	down
36	SDC1	5.23E-03	1.98	0.98	up
37	GLIPR2	5.28E-03	1.30	0.38	up
38	PDGFRB	5.30E-03	1.56	0.64	up
39	MASP2	5.30E-03	0.82	-0.28	down
40	CDH5	5.65E-03	1.29	0.37	up
41	ABI3BP	5.65E-03	1.36	0.44	up
42	S100A8	5.67E-03	0.74	-0.44	down
43	KLHL36	5.95E-03	0.74	-0.44	down
44	ORM1	6.45E-03	0.75	-0.41	down
45	HIST1H1E	6.55E-03	0.48	-1.05	down
46	PPP1R15A	6.88E-03	0.74	-0.43	down
47	NTM	7.28E-03	1.90	0.93	up
48	LYVE1	7.30E-03	1.24	0.31	up
49	LYZ	7.33E-03	1.20	0.27	up
50	IGHV5-51	7.48E-03	0.63	-0.66	down
51	NRP1	7.53E-03	1.21	0.28	up

52	ATP1A1	7.75E-03	0.77	-0.37	down
53	PLXDC2	8.10E-03	1.21	0.28	up
54	NPC2	8.14E-03	1.30	0.38	up
55	PLOD1	8.17E-03	1.35	0.43	up
56	APOC3	8.28E-03	0.68	-0.55	down
57	HEG1	8.61E-03	1.33	0.41	up
58	MAP4	8.88E-03	1.51	0.60	up
59	PRAM1	9.20E-03	0.55	-0.86	down
60	CD84	9.57E-03	1.56	0.64	up
61	SERPINA6	9.88E-03	1.21	0.28	up
62	LBP	9.92E-03	0.70	-0.51	down
63	ADA2	1.12E-02	1.33	0.41	up
64	CNDP1	1.14E-02	0.76	-0.40	down
65	PODXL	1.19E-02	1.23	0.30	up
66	MAPK1	1.20E-02	0.76	-0.40	down
67	F12	1.21E-02	0.76	-0.39	down
68	NAP1L4	1.23E-02	1.50	0.59	up
69	VNN1	1.28E-02	0.61	-0.72	down
70	IGLV1-40	1.28E-02	0.43	-1.22	down
71	PRDX5	1.30E-02	1.73	0.79	up
72	GDF2	1.37E-02	0.35	-1.53	down
73	PKHD1L1	1.37E-02	1.95	0.96	up
74	REG1A	1.40E-02	1.45	0.54	up
75	IGLV2-18	1.54E-02	0.55	-0.86	down
76	PSMA1	1.57E-02	1.67	0.74	up
77	NEO1	1.66E-02	1.24	0.31	up
78	HEXA	1.66E-02	1.57	0.65	up
79	SERPINA11	2.09E-02	1.34	0.42	up
80	HERC4	2.10E-02	0.46	-1.13	down
81	MDH2	2.19E-02	2.30	1.20	up
82	GRK3	2.25E-02	0.70	-0.52	down
83	KIT	2.29E-02	1.27	0.35	up
84	C1QTNF3	2.32E-02	1.24	0.31	up
85	ST3GAL6	2.34E-02	1.89	0.92	up
86	HSP90B1	2.38E-02	1.23	0.30	up
87	CXCL12	2.38E-02	0.06	-4.13	down
88	SECTM1	2.41E-02	0.69	-0.53	down
89	TTN	2.47E-02	1.27	0.34	up
90	IGLV8-61	2.48E-02	0.61	-0.72	down
91	CST6	2.60E-02	1.32	0.40	up
92	SLC3A2	2.62E-02	1.22	0.28	up
93	ADSSL1	2.63E-02	0.70	-0.51	down
94	IGHV3OR16-12	2.70E-02	0.69	-0.53	down
95	AHNAK	2.72E-02	0.70	-0.51	down
96	COL15A1	2.90E-02	1.32	0.40	up
97	ROBO4	2.90E-02	1.30	0.38	up
98	MGP	2.99E-02	1.45	0.53	up
99	IGLV6-57	3.02E-02	0.20	-2.31	down
100	DSG2	3.07E-02	1.35	0.43	up
101	FTL	3.11E-02	0.39	-1.37	down
102	IGHV3OR16-13	3.23E-02	0.73	-0.44	down
103	MADCAM1	3.24E-02	0.57	-0.80	down
104	PRG4	3.32E-02	0.75	-0.41	down
105	SLC2A1	3.35E-02	2.06	1.04	up

106	ACE	3.46E-02	1.24	0.31	up
107	ELANE	3.48E-02	0.52	-0.93	down
108	RNASET2	3.52E-02	3.47	1.80	up
109	GFAP	3.54E-02	0.68	-0.56	down
110	MIA3	3.56E-02	2.23	1.16	up
111	HNRNPA1	3.59E-02	2.41	1.27	up
112	IGKV1-27	3.61E-02	0.41	-1.27	down
113	LGALS3BP	3.63E-02	0.81	-0.30	down
114	FLNA	3.64E-02	1.34	0.42	up
115	PCDH1	3.65E-02	0.47	-1.09	down
116	SPP1	3.68E-02	1.91	0.94	up
117	GNPNAT1	3.70E-02	1.57	0.65	up
118	HSP90AA1	3.73E-02	1.59	0.67	up
119	SOD3	3.79E-02	1.22	0.28	up
120	CD109	3.80E-02	1.33	0.41	up
121	ATP5F1B	3.82E-02	3.67	1.87	up
122	HSPA9	3.91E-02	1.62	0.70	up
123	SAA4	3.92E-02	0.80	-0.31	down
124	PDIA4	3.96E-02	2.05	1.04	up
125	CRIP1	3.99E-02	1.34	0.43	up
126	UGP2	4.04E-02	0.50	-1.01	down
127	ST8SIA4	4.05E-02	2.54	1.35	up
128	PI3	4.06E-02	1.55	0.63	up
129	GSN	4.07E-02	1.35	0.44	up
130	SPTLC2	4.15E-02	1.33	0.41	up
131	APOC2	4.15E-02	0.67	-0.57	down
132	MTPN	4.19E-02	2.25	1.17	up
133	APOC4	4.21E-02	0.62	-0.69	down
134	MGRN1	4.32E-02	2.37	1.25	up
135	NOTCH2	4.40E-02	1.20	0.27	up
136	PPP2R1A	4.44E-02	6.34	2.66	up
137	SERPINB12	4.53E-02	2.94	1.56	up
138	FAM172A	4.55E-02	0.73	-0.45	down
139	ACSBG1	4.56E-02	0.78	-0.36	down
140	CETP	4.61E-02	1.35	0.44	up
141	LSR	4.62E-02	4.22	2.08	up
142	EXT1	4.80E-02	1.33	0.41	up
143	XPNPEP3	4.80E-02	0.65	-0.62	down
144	OGN	4.92E-02	1.35	0.43	up
145	HIST1H2BD	4.98E-02	1.24	0.31	up
146	NCAM2	4.98E-02	1.51	0.60	up
