# Supplementary material for: Multi-Omics Mechanism of Chronic Gout Arthritis and Discovery of the Thyroid Hormone–AMPK–Taurine Metabolic Axis
Source: Cells. 2025 Dec 25;15(1):41. doi: 10.3390/cells15010041 (PMC12785424; doi:10.3390/cells15010041)
Supplement: Supplementary file 1 [file cells-15-00041-s001.zip › Differentially expressed proteins in CGA vs control comparison.pdf]

Differentially expressed proteins in CGA vs control comparison

No.	Protein	P	FC	log2FC	Up.Down
1	F9	3.05E-10	2.03	1.02	up
2	LBP	4.00E-08	1.98	0.98	up
3	GPX3	1.72E-06	0.77	-0.37	down
4	EEF2	2.28E-06	0.44	-1.18	down
5	F11	2.90E-06	2.03	1.02	up
6	GUCY1A2	4.52E-06	0.39	-1.37	down
7	CD14	8.88E-06	1.56	0.64	up
8	ANG	8.91E-06	1.51	0.59	up
9	C9	1.24E-05	1.95	0.96	up
10	FGA	1.55E-05	0.58	-0.79	down
11	F7	3.26E-05	1.37	0.45	up
12	INHBC	4.40E-05	1.74	0.80	up
13	SMOC1	4.91E-05	0.31	-1.70	down
14	PROS1	5.20E-05	1.23	0.30	up
15	APOA2	5.89E-05	0.79	-0.34	down
16	XPNPEP3	7.80E-05	0.41	-1.27	down
17	IL1R2	7.88E-05	0.62	-0.70	down
18	C1QA	8.33E-05	1.21	0.27	up
19	QSOX1	8.59E-05	1.49	0.58	up
20	LYZ	8.69E-05	1.30	0.38	up
21	TTR	1.14E-04	0.76	-0.39	down
22	CST3	1.43E-04	1.23	0.30	up
23	FAM135B	1.55E-04	1.63	0.71	up
24	HLA-B	1.74E-04	0.48	-1.06	down
25	SERPINA5	1.99E-04	1.69	0.76	up
26	ZBTB20	2.10E-04	1.77	0.82	up
27	PRG4	2.23E-04	1.52	0.60	up
28	SPARCL1	3.45E-04	0.80	-0.31	down
29	EIF2S2	3.65E-04	0.30	-1.74	down
30	TREML1	5.07E-04	0.49	-1.03	down
31	EEF1G	6.29E-04	0.27	-1.90	down
32	PRAP1	8.25E-04	1.48	0.57	up
33	EFEMP1	9.52E-04	1.96	0.97	up
34	SERPIND1	1.00E-03	1.67	0.74	up
35	SEMA4D	1.07E-03	0.58	-0.79	down
36	FETUB	1.09E-03	1.26	0.33	up
37	SERPINF2	1.23E-03	1.40	0.49	up
38	GGT1	1.25E-03	2.31	1.21	up
39	IGHV3-7	1.27E-03	0.66	-0.61	down
40	IGKV3-20	1.86E-03	0.68	-0.55	down
41	FLNA	1.89E-03	0.64	-0.64	down
42	ARR3	2.02E-03	1.30	0.38	up
43	RPL3	2.38E-03	0.39	-1.35	down
44	IGKV1-5	2.64E-03	0.68	-0.55	down
45	NOMO3	2.64E-03	0.80	-0.32	down
46	ALB	2.69E-03	0.37	-1.44	down
47	PRSS3	2.83E-03	1.77	0.83	up
48	PCDH1	2.86E-03	0.36	-1.47	down
49	SRGN	3.04E-03	0.75	-0.41	down
50	ITIH3	3.32E-03	1.46	0.55	up
51	C8A	3.44E-03	1.43	0.52	up

52	IGKV1-33	3.76E-03	0.77	-0.38	down
53	ITIH1	3.84E-03	1.45	0.53	up
54	ITIH4	3.90E-03	1.26	0.34	up
55	PMS1	3.96E-03	1.44	0.52	up
56	IGHV3OR16-12	3.97E-03	0.58	-0.78	down
57	IGKV3D-11	4.27E-03	0.67	-0.58	down
58	CFHR5	4.52E-03	1.36	0.45	up
59	ACLY	4.92E-03	0.44	-1.17	down
60	C8G	5.18E-03	1.46	0.54	up
61	C1S	5.49E-03	1.21	0.28	up
62	VASN	5.71E-03	1.25	0.33	up
63	IGKV1-8	5.92E-03	0.45	-1.16	down
64	IGHV3-30	5.98E-03	0.69	-0.54	down
65	IGKC	6.10E-03	0.74	-0.43	down
66	IGKV2D-29	6.40E-03	0.67	-0.58	down
67	PPP1R15A	7.01E-03	0.72	-0.48	down
68	COMP	7.52E-03	1.26	0.34	up
69	MYO15B	7.64E-03	1.29	0.36	up
70	C5	8.12E-03	1.38	0.47	up
71	CNDP1	8.64E-03	0.73	-0.45	down
72	RPS6KC1	8.70E-03	1.27	0.35	up
73	HNRNPU	8.73E-03	0.69	-0.54	down
74	NCAM1	9.05E-03	0.82	-0.29	down
75	SERPINA4	9.21E-03	1.48	0.56	up
76	MAN1A1	9.26E-03	1.23	0.30	up
77	AHNAK2	9.33E-03	1.37	0.45	up
78	F12	9.37E-03	0.73	-0.46	down
79	C1R	9.74E-03	1.26	0.33	up
80	CDH5	1.06E-02	1.28	0.35	up
81	ITIH2	1.08E-02	1.37	0.45	up
82	ACAN	1.13E-02	0.79	-0.33	down
83	IGHV3OR16-13	1.13E-02	0.67	-0.58	down
84	WDR1	1.17E-02	0.59	-0.77	down
85	LSAMP	1.18E-02	0.74	-0.43	down
86	IGKV1D-39	1.19E-02	0.67	-0.59	down
87	IGHG1	1.31E-02	0.76	-0.40	down
88	GNPNAT1	1.40E-02	2.11	1.08	up
89	FBLN1	1.44E-02	1.69	0.75	up
90	GSN	1.45E-02	1.48	0.56	up
91	SELP	1.47E-02	0.70	-0.50	down
92	CPN2	1.49E-02	1.20	0.27	up
93	GINM1	1.53E-02	1.70	0.77	up
94	MAN1C1	1.55E-02	1.45	0.54	up
95	AMY1B	1.55E-02	0.78	-0.35	down
96	S100A9	1.57E-02	0.35	-1.50	down
97	ARHGDIB	1.63E-02	0.50	-1.01	down
98	ANKRD46	1.65E-02	1.26	0.33	up
99	MSLN	1.68E-02	1.41	0.50	up
100	NT5DC2	1.71E-02	1.44	0.53	up
101	TPM1	1.88E-02	0.57	-0.81	down
102	MGP	1.91E-02	1.63	0.70	up
103	OAF	1.93E-02	1.20	0.26	up
104	SLIT1	1.94E-02	0.42	-1.25	down
105	MYBPH	1.94E-02	1.26	0.34	up

106	SLC2A8	1.97E-02	0.28	-1.86	down
107	COL6A3	2.01E-02	1.21	0.27	up
108	PCDH18	2.02E-02	0.29	-1.77	down
109	ANXA4	2.07E-02	0.20	-2.32	down
110	HLA-A	2.18E-02	0.62	-0.68	down
111	SHBG	2.38E-02	1.88	0.91	up
112	IGHV5-51	2.38E-02	0.72	-0.48	down
113	MIF	2.38E-02	0.28	-1.86	down
114	MMP2	2.40E-02	1.25	0.32	up
115	TCN2	2.51E-02	2.11	1.07	up
116	IGLV2-18	2.67E-02	0.30	-1.75	down
117	C8B	2.72E-02	1.45	0.53	up
118	PDS5B	2.78E-02	1.31	0.38	up
119	IGHV3OR15-7	2.82E-02	0.60	-0.73	down
120	IGLV4-69	2.87E-02	0.30	-1.71	down
121	ADIPOQ	2.91E-02	0.74	-0.43	down
122	CD84	2.96E-02	1.45	0.54	up
123	IGLV9-49	3.00E-02	0.45	-1.14	down
124	FN1	3.06E-02	2.16	1.11	up
125	CD99	3.09E-02	1.37	0.45	up
126	GLIPR2	3.23E-02	1.24	0.31	up
127	IGHV3-35	3.23E-02	0.74	-0.43	down
128	FAM172A	3.26E-02	0.74	-0.43	down
129	PDGFRB	3.44E-02	1.40	0.49	up
130	DBNL	3.46E-02	0.20	-2.35	down
131	F2	3.46E-02	1.36	0.44	up
132	FAM135A	3.52E-02	0.78	-0.36	down
133	TUBB1	3.61E-02	0.19	-2.41	down
134	DKK3	3.67E-02	0.73	-0.45	down
135	IGHV3-43	3.67E-02	0.61	-0.71	down
136	PFN1	3.78E-02	0.51	-0.97	down
137	TKT	3.87E-02	0.71	-0.49	down
138	PSMA3	3.90E-02	0.60	-0.74	down
139	CD209	3.92E-02	1.46	0.55	up
140	SH3BGRL3	3.96E-02	0.61	-0.71	down
141	GOT1	4.04E-02	0.77	-0.38	down
142	HSPA9	4.09E-02	0.68	-0.56	down
143	TAGLN2	4.12E-02	0.37	-1.44	down
144	NEO1	4.30E-02	1.20	0.27	up
145	NUCB1	4.49E-02	0.78	-0.36	down
146	APEH	4.60E-02	0.59	-0.75	down
147	HSPD1	4.61E-02	0.78	-0.37	down
148	CDH17	4.66E-02	1.38	0.47	up
149	PKHD1L1	4.69E-02	1.59	0.67	up
150	LCP1	4.71E-02	1.33	0.41	up
151	TPM3	4.85E-02	0.46	-1.12	down
152	JCHAIN	4.91E-02	0.73	-0.46	down
153	DGKH	4.92E-02	0.80	-0.33	down
154	GFAP	4.92E-02	0.53	-0.92	down
