# Supplementary material for: Multi-Omics Mechanism of Chronic Gout Arthritis and Discovery of the Thyroid Hormone–AMPK–Taurine Metabolic Axis
Source: Cells. 2025 Dec 25;15(1):41. doi: 10.3390/cells15010041 (PMC12785424; doi:10.3390/cells15010041)
Supplement: Supplementary file 1 [file cells-15-00041-s001.zip › Revised supplementary.pdf]

Multi-Omics Mechanism of Chronic Gout Arthritis and Discovery of the Thyroid Hormone-AMPK-Taurine Metabolic Axis

Guizhen Zhu [1,†], Yuan Luo [1,†], Xiangyi Zheng [1], Zhusong Mei [1], Qiao Ye [1], Jie Peng [1], Fengsen Duan [1], Yueying Cui [1], Peiyu An [1], Yangqian Song [2], Hongxia Li [2], Haitao Zhang [3,*], Guangyun Wang [1,*]

[1] Laboratory of Clinical Medicine, Air Force Medical Center, Air Force Medical University, People's Liberation Army of China, Beijing 100142, China

[2] Rheumatology and Immunology Department, Air Force Medical Center, Air Force Medical University, People's Liberation Army of China, Beijing 100142, China

[3] Cardiovascular Department, Air Force Medical Center, Air Force Medical University, People's Liberation Army of China, Beijing 100142, China

* Correspondence: kjzht@sina.com (H.Z.); gfkdwgy@163.com (G.W.)

† These authors contributed equally to this work.

Supporting Figures

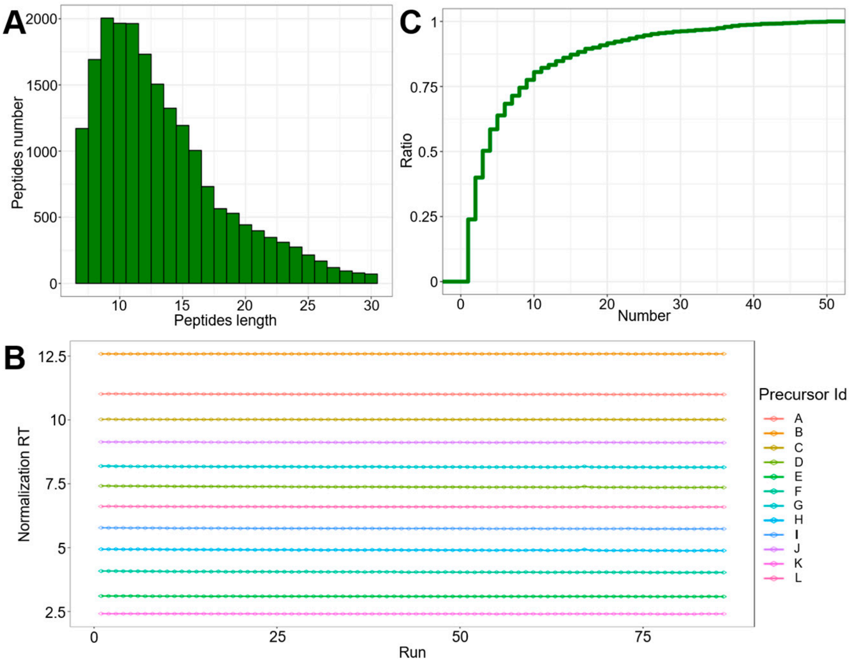

Figure S1 Quality control analysis of DIA-based quantitative proteomic data. (A) Peptide length range distribution plot. (B) iRT values of internal standard calibrated peptides. A-L represent the internal standard calibrated peptides. (C) Distribution plot of unique peptide counts in identified proteins.

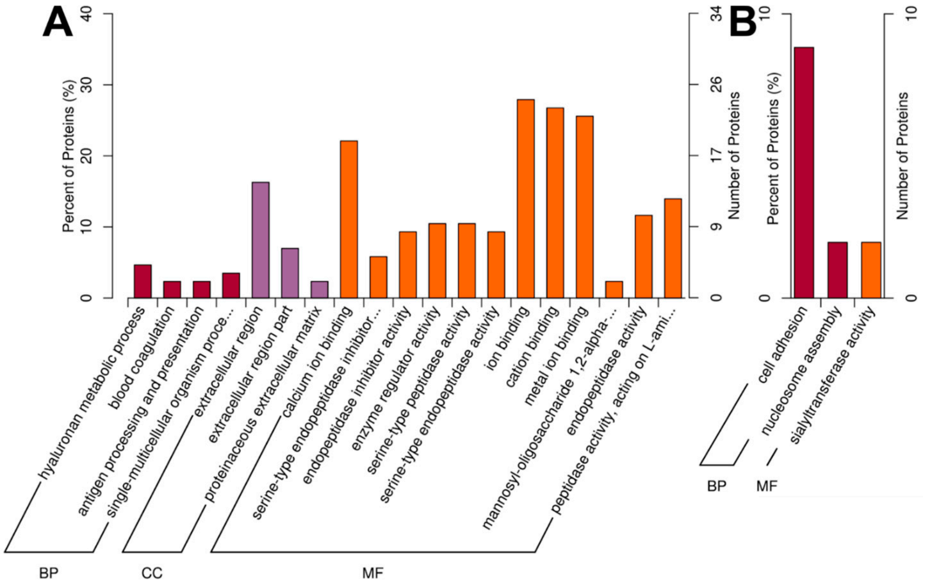

Figure S2 Functional enrichment analysis of GO with statistical significance for differential proteins in (A) CGA vs control and (B) CGA vs AGA groups.

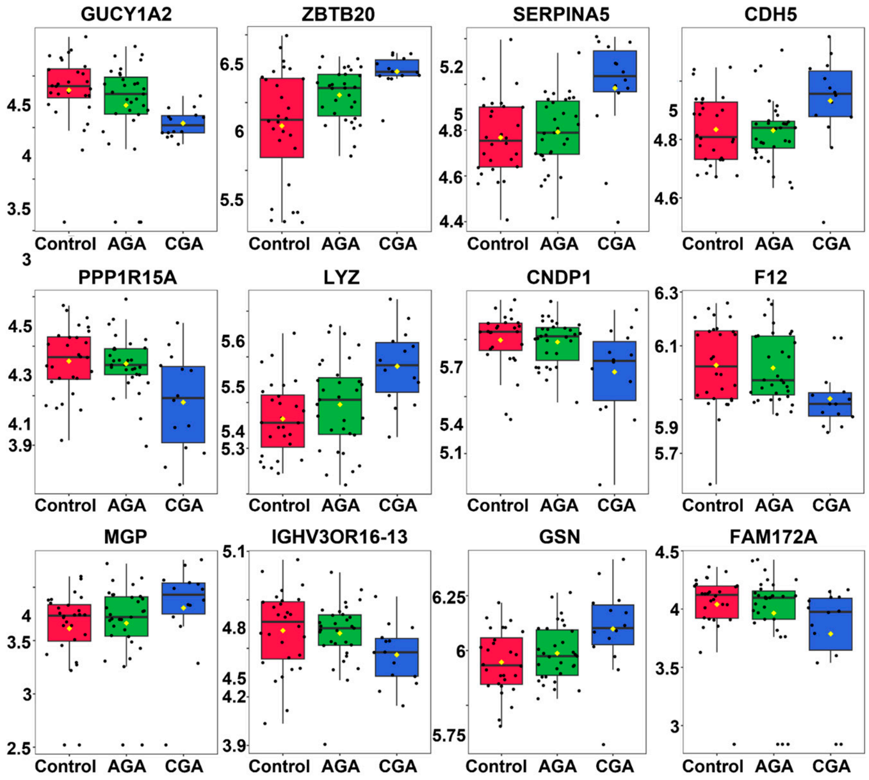

Figure S3 Box plot analysis of differential proteins exhibiting consistent expression trends across control, AGA, and CGA groups.

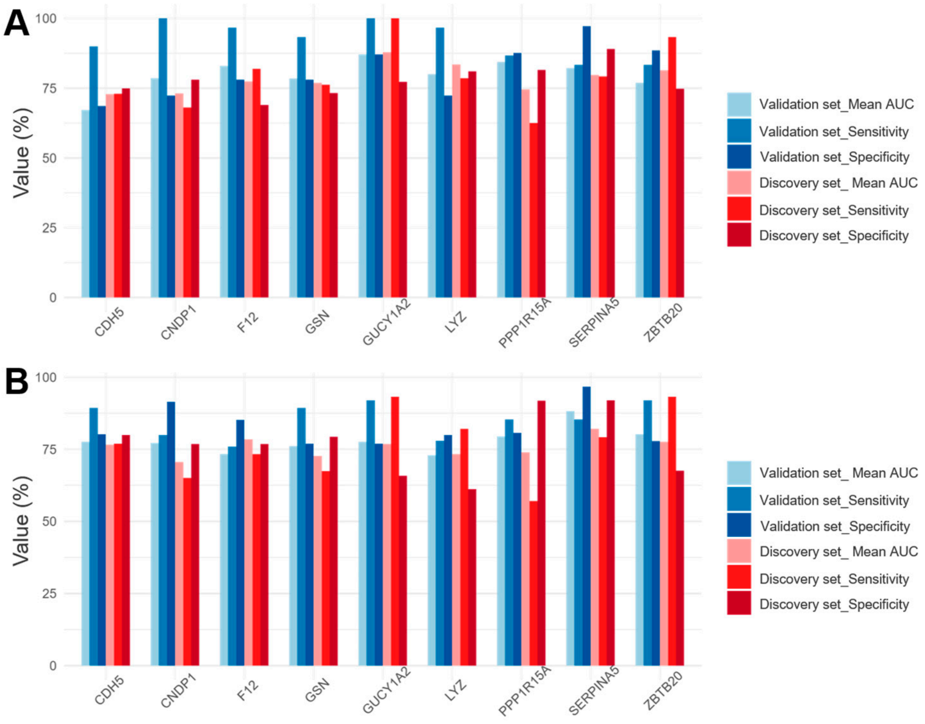

Figure S4 Results of the 5-fold cross-validation for the core proteins in the CAG vs control and CAG vs AGA comparisons across the discovery and validation sets.

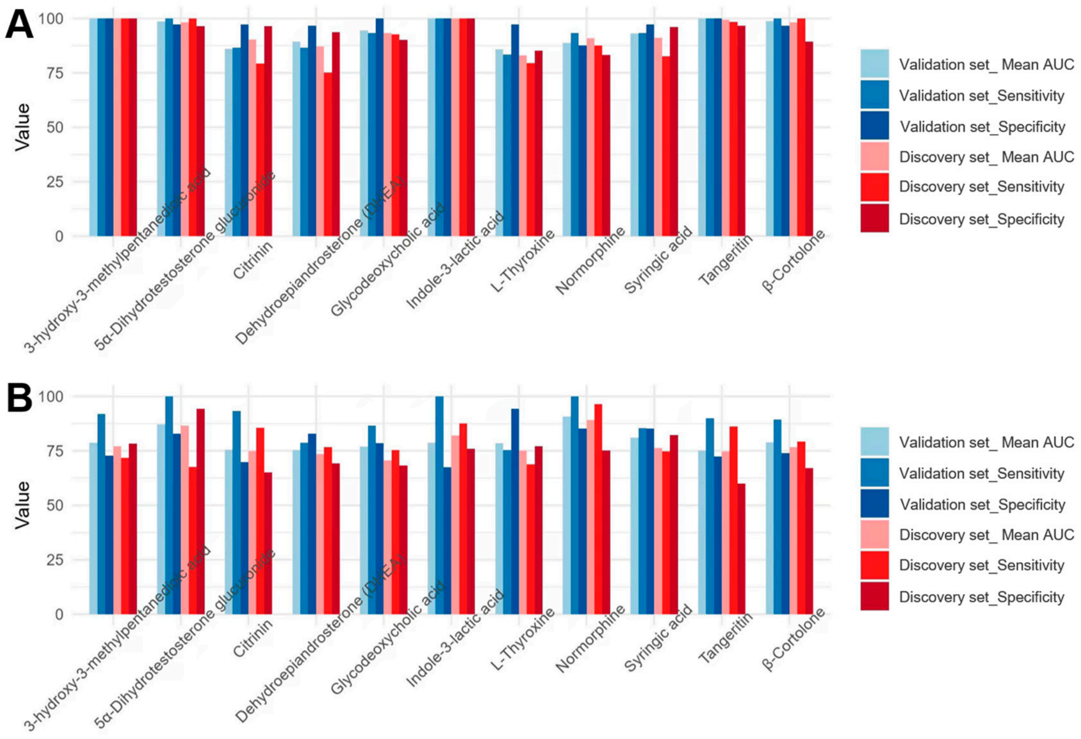

Figure S5 Results of the 5-fold cross-validation for the core metabolites in the CAG vs control and CAG vs AGA comparisons across the discovery and validation sets.

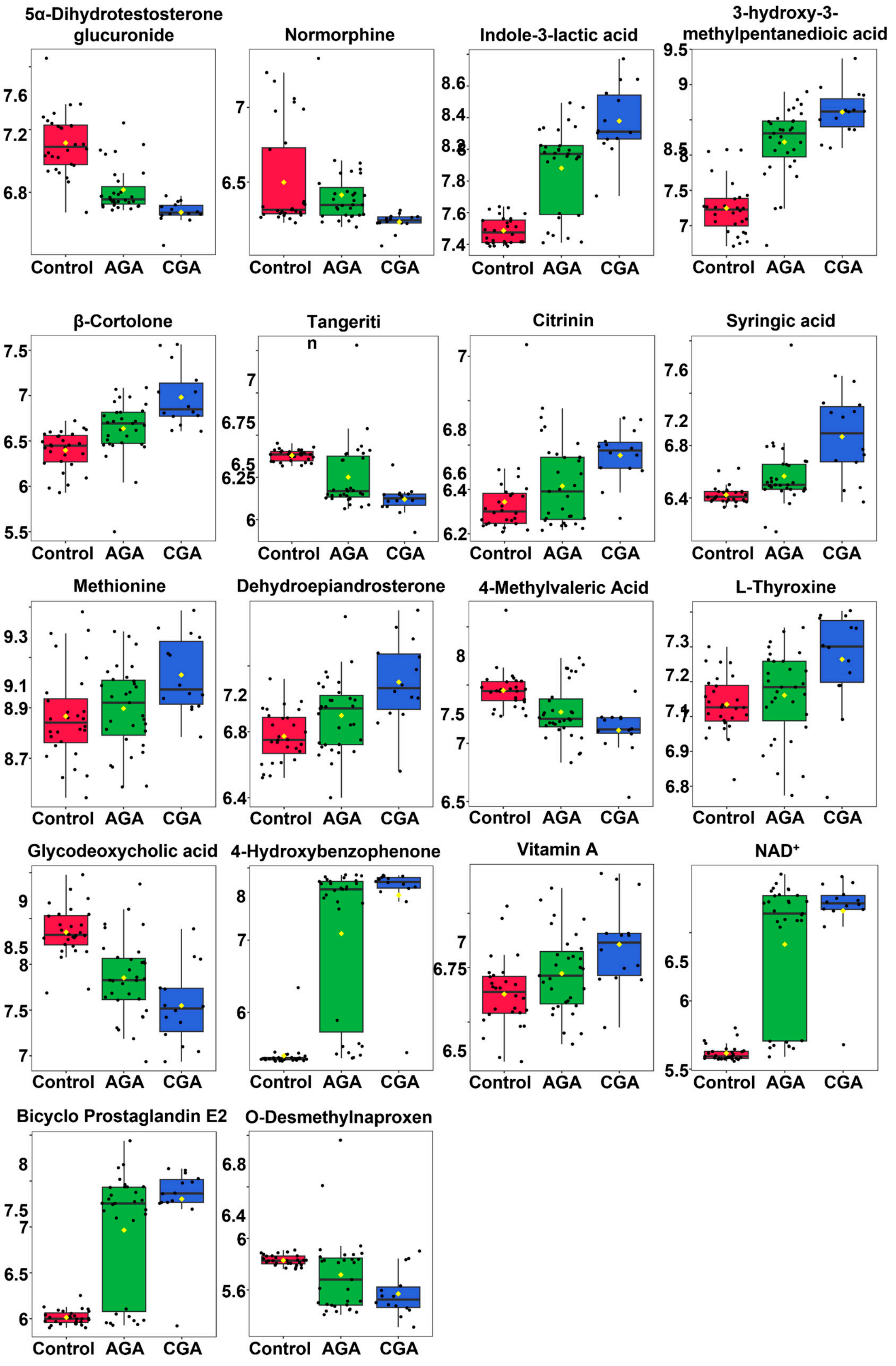

Figure S6 Box plot analysis of differential metabolites exhibiting consistent trends across control, AGA, and CGA groups with annotations in HMDB, KEGG, and LipidMaps databases.

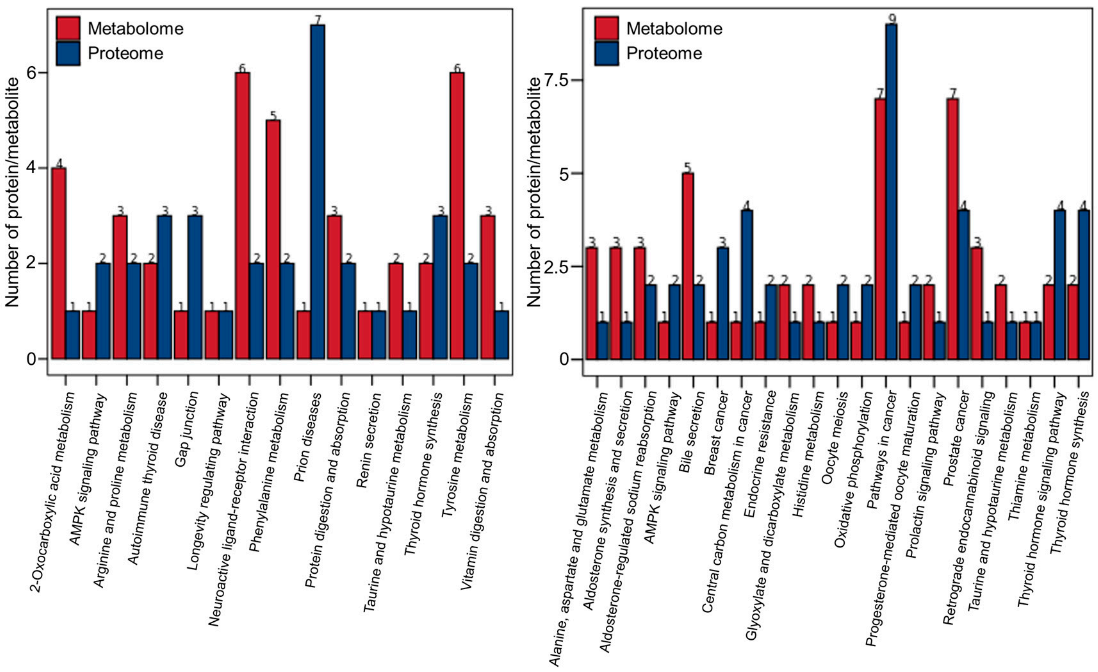

Figure S7 Quantification of differentially expressed proteins and metabolites in pathways commonly enriched in (A) CGA vs control and (B) CGA vs AGA comparisons.

Supporting Tables

Table S1 EGG pathway enrichment analysis of differentially expressed proteins and differential metabolites in CGA vs control comparison.

No.	Pathway	Ratio	Pvalue	Count	Type
1	Tyrosine metabolism	0.14	1.81E-01	2	Proteomics
		0.75	1.47E-03	6	Metabolomics
2	Neuroactive ligand-receptor interaction	0.25	6.90E-02	2	Proteomics
		0.50	2.34E-02	6	Metabolomics
3	Autoimmune thyroid disease	0.12	1.59E-01	3	Proteomics
		1.00	4.48E-02	2	Metabolomics
4	Thyroid hormone synthesis	0.17	7.42E-02	3	Proteomics
		0.67	1.16E-01	2	Metabolomics
5	Prion diseases	0.32	1.11E-04	7	Proteomics
		1.00	2.13E-01	1	Metabolomics
6	Longevity regulating pathway	0.09	4.69E-01	1	Proteomics
		1.00	2.13E-01	1	Metabolomics
7	Phenylalanine metabolism	0.33	3.97E-02	2	Proteomics
		0.33	3.26E-01	5	Metabolomics
8	Renin secretion	0.10	4.37E-01	1	Proteomics
		0.50	3.81E-01	1	Metabolomics

9	AMPK signaling pathway	0.10	3.29E-01	2	Proteomics
		0.50	3.81E-01	1	Metabolomics
10	Vitamin digestion and absorption	0.17	2.91E-01	1	Proteomics
		0.30	4.48E-01	3	Metabolomics
11	2-Oxocarboxylic acid metabolism	0.10	4.37E-01	1	Proteomics
		0.29	5.06E-01	4	Metabolomics
12	Taurine and hypotaurine metabolism	0.50	1.08E-01	1	Proteomics
		0.33	6.11E-01	2	Metabolomics
13	Protein digestion and absorption	0.08	6.46E-01	2	Proteomics
		0.25	7.23E-01	3	Metabolomics
14	Gap junction	0.12	1.73E-01	3	Proteomics
		0.25	1.00E+00	1	Metabolomics
15	Arginine and proline metabolism	0.11	2.65E-01	2	Proteomics
		0.23	1.00E+00	3	Metabolomics

Table S2 EGG pathway enrichment analysis of differentially expressed proteins and differential metabolites in CGA vs AGA comparison.

No.	Description	Ratio	Pvalue	Count	Type
1	Prostate cancer	0.19	2.92E-02	4	Proteomics
		0.88	3.97E-05	7	Metabolomics
2	Pathways in cancer	0.10	1.08E-01	9	Proteomics
		0.54	3.87E-03	7	Metabolomics
3	Aldosterone-regulated sodium reabsorption	0.29	5.74E-02	2	Proteomics
		1.00	6.31E-03	3	Metabolomics
4	Thyroid hormone signaling pathway	0.22	1.71E-02	4	Proteomics
		1.00	3.47E-02	2	Metabolomics
5	Thyroid hormone synthesis	0.22	1.71E-02	4	Proteomics
		0.67	9.15E-02	2	Metabolomics
6	Prolactin signaling pathway	0.10	4.50E-01	1	Proteomics
		0.67	9.15E-02	2	Metabolomics
7	Aldosterone synthesis and secretion	0.11	4.16E-01	1	Proteomics
		0.43	1.25E-01	3	Metabolomics
8	Alanine, aspartate and glutamate metabolism	0.07	5.67E-01	1	Proteomics
		0.43	1.25E-01	3	Metabolomics
9	Progesterone-mediated oocyte maturation	0.25	7.37E-02	2	Proteomics
		1.00	1.88E-01	1	Metabolomics
10	Oocyte meiosis	0.12	2.58E-01	2	Proteomics

		1.00	1.88E-01	1	Metabolomics
11	Taurine and hypotaurine metabolism	0.50	1.12E-01	1	Proteomics
		0.33	3.14E-01	2	Metabolomics
12	Oxidative phosphorylation	0.07	6.67E-01	2	Proteomics
		0.50	3.41E-01	1	Metabolomics
13	Endocrine resistance	0.15	1.71E-01	2	Proteomics
		0.50	3.41E-01	1	Metabolomics
14	Breast cancer	0.20	5.12E-02	3	Proteomics
		0.50	3.41E-01	1	Metabolomics
15	AMPK signaling pathway	0.10	3.45E-01	2	Proteomics
		0.50	3.41E-01	1	Metabolomics
16	Thiamine metabolism	0.25	2.12E-01	1	Proteomics
		0.33	4.65E-01	1	Metabolomics
17	Histidine metabolism	0.10	4.50E-01	1	Proteomics
		0.29	6.19E-01	2	Metabolomics
18	Glyoxylate and dicarboxylate metabolism	0.06	6.16E-01	1	Proteomics
		0.25	6.47E-01	2	Metabolomics
19	Bile secretion	0.40	2.95E-02	2	Proteomics
		0.20	7.93E-01	5	Metabolomics
20	Retrograde endocannabinoid signaling	0.08	5.12E-01	1	Proteomics
		0.20	1.00E+00	3	Metabolomics
21	Central carbon metabolism in cancer	0.20	2.47E-02	4	Proteomics
		0.20	1.00E+00	1	Metabolomics
